# Data-driven modeling and prediction of non-linearizable dynamics via spectral submanifolds

**DOI:** 10.1038/s41467-022-28518-y

**Published:** 2022-02-15

**Authors:** Mattia Cenedese, Joar Axås, Bastian Bäuerlein, Kerstin Avila, George Haller

**Affiliations:** 1 Institute for Mechanical Systems, ETH Zürich, Leonhardstrasse 21, 8092 Zürich, Switzerland; 2 University of Bremen, Faculty of Production Engineering, Badgasteiner Strasse 1, 28359 Bremen, Germany; 3 Leibniz Institute for Materials Engineering IWT, Badgasteiner Strasse 3, 28359 Bremen, Germany

**Keywords:** Scientific data, Mechanical engineering

## Abstract

We develop a methodology to construct low-dimensional predictive models from data sets representing essentially nonlinear (or *non-linearizable*) dynamical systems with a hyperbolic linear part that are subject to external forcing with finitely many frequencies. Our data-driven, sparse, nonlinear models are obtained as extended normal forms of the reduced dynamics on low-dimensional, attracting spectral submanifolds (SSMs) of the dynamical system. We illustrate the power of data-driven SSM reduction on high-dimensional numerical data sets and experimental measurements involving beam oscillations, vortex shedding and sloshing in a water tank. We find that SSM reduction trained on unforced data also predicts nonlinear response accurately under additional external forcing.

## Abstract (web summary)

Current data-driven modelling techniques perform reliably on linear systems or on those that can be linearized. Cenedese et al. develop a data-based reduced modeling method for non-linear, high-dimensional physical systems. Their models reconstruct and predict the dynamics of the full physical system.

## Introduction

Low-dimensional reduced models of high-dimensional nonlinear dynamical systems are critically needed in various branches of applied science and engineering. Such simplified models would significantly reduce computational costs and enable physical interpretability, design optimization and efficient controllability. As of yet, however, no generally applicable procedure has emerged for the reliable and robust identification of nonlinear reduced models.

Instead, the most broadly used approach to reducing nonlinear dynamical systems has been a fundamentally linear technique, the proper orthogonal decomposition (POD), followed by a Galerkin projection^1–3^. Projecting the full dynamics to the most energetic linear modes, POD requires the knowledge of the governing equations of the system and hence is inapplicable when only data is available. As purely data-based alternatives, machine learning methods are broadly considered and tested in various fields^4–7^. While the black-box approach of machine learning might often seem preferable to a detailed nonlinear analysis, the resulting neural network models require extensive tuning, lack physical interpretability, generally perform poorly outside their training range and tend to be unnecessarily complex^8^. This has inspired a number of approaches that seek a blend of machine learning with a priori information about the underlying physics^9,10^. Still within the realm of machine learning, sparse regression has also shown promise in approximating the right-hand sides of low-dimensional, simple dynamical systems with functions taken from a preselected library^4^. Another recent approach is cluster-based network modeling, which uses the toolkit of network science and statistical physics for modeling nonlinear dynamics^11^.

A popular alternative to POD and machine learning is the dynamic mode decomposition (DMD)^12^, which approximates directly the observed system dynamics. The original DMD and its later variants fit a linear dynamical system to temporally evolving data, possibly including further functions of the original data, over a given finite time interval^13^. DMD provides an appealingly simple yet powerful algorithm to infer a local model near steady states where the nonlinear dynamics is always approximately linear. This linear model is also more globally valid if constructed over observables lying in a span of some eigenfunctions of the Koopman operator, which maps observables evaluated over initial states into their evaluations over current states^14–16^. This relationship between DMD and the Koopman operator has motivated an effort to machine-learn Koopman eigenfunctions from data in order to linearize nonlinear dynamical systems globally on the space of their observables^17–19^.

Finding physically realizable observables that fall in a Koopman eigenspace is, however, often described as challenging or difficult^20^. A more precise assessment would be that such a find is highly unlikely, given that the probability of any countable set of a priori selected observables falling in any Koopman eigenspace is zero. In addition, those eigenspaces can only be determined explicitly in simple, low-dimensional systems. In practice, therefore, DMD can only provide a justifiable model near an attracting fixed point of a dynamical system. While Koopman modes still have the potential to linearize the observer dynamics on larger domains, those domains cannot include more than one attracting or repelling fixed point^19–21^. Indeed, DMD and Koopman mode expansions fail to converge outside neighborhoods of fixed points even in the simplest, one-dimensional nonlinear systems with two fixed points^20,22^. In summary, while these data-driven model reduction methods are powerful and continue to inspire ongoing research, their applicability is limited to locally linearized systems and globally linearizable nonlinear systems, such as the Burgers equation^23^.

The focus of this paper is the development of data-driven, simple and predictive reduced-order models for essentially nonlinear dynamical systems, i.e., nonlinearizable systems. Determining exact linearizability conclusively from data is beyond reach. In contrast, establishing that a dynamical system is nonlinearizable in a domain of interest is substantially simpler: one just needs to find an indication of coexisting isolated stationary states in the data. By an isolated stationary state, we mean here a compact and connected invariant set with an open neighborhood that contains no other compact and connected invariant set. Examples of such stationary states include hyperbolic fixed points, periodic orbits, invariant spheres and quasiperiodic tori; closures of homoclinic orbits and heteroclinic cycles; and chaotic attractors and repellers. If a data set indicates the coexistence of any two sets from the above list, then the system is conclusively non-linearizable in the range of the available data. Specifically, there will be no homeomorphism (continuous transformation with a continuous inverse) that transforms the orbits of the underlying dynamical system into those of a linear dynamical system. While this is a priori clear from dynamical systems theory, several studies have specifically confirmed a lack of convergence of Koopman-mode expansions already for the simplest case of two coexisting fixed points, even over subsets of their domain of attraction or repulsion^20,22^.

**Fig. 1:**
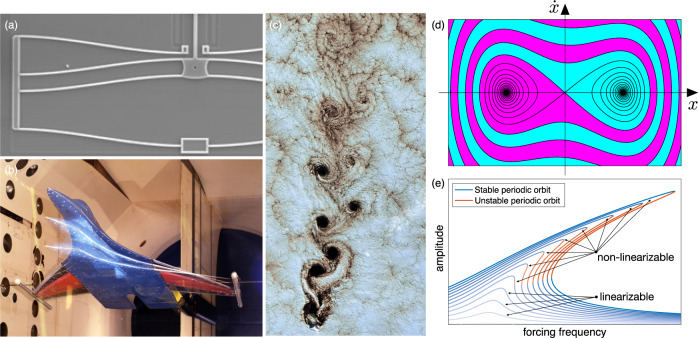
Examples of non-linearizable systems. **a** Snap-through instability of a microelectro-mechanical (MEMS) device with three coexisting equilibria (Sandia National Laboratories). **b** Wind-tunnel flutter of an airplane prototype, involving a fixed point and coexisting limit cycles (NASA Langley Research Center). **c** Swirling clouds behind an island in the Pacific ocean, forming a vortex street with coexisting isolated hyperbolic and elliptic trajectories for the dynamical system describing fluid particle motion (USGS/NASA). **d** Phase portrait of the damped, double-well Duffing oscillator $\ddot{x}+\dot{x}-x+\beta {x}^{3}=0$ with *β* > 0, the most broadly used model for nonlinear systems with coexisting domains of attraction (colored), such as the MEMS device in plot (**a**). **e** Nonlinear response amplitude ($| x(t){| }_{\max }$) in the forced-damped, single-well Duffing oscillator, $\ddot{x}+\dot{x}+x+\beta {x}^{3}=f\cos \omega t$ with *β* > 0, under variations of the forcing frequency *ω* and forcing amplitude *f*. Coexisting stable and unstable periodic responses show non-linearizable dynamics conclusively for this classic model.

Non-linearizable systems are ubiquitous in science, technology and nature. Beyond the well-known examples of chaotic dynamical systems and turbulent fluid flows^1^, any bifurcation phenomenon, by definition, involves coexisting steady states and hence is automatically non-linearizable. Indeed, aerodynamic flutter^24^, buckling of beams and shells^25^, bistable microelectromechanical systems^26^, traffic jams^27^ or even tipping points in climate change^28^ are all fundamentally non-linearizable, just to name a few. Figure 1 shows some examples of non-linearizable systems emerging in technology, nature and scientific modeling.

We will show here that a collection of classic and recent mathematical results from nonlinear dynamical systems theory enables surprisingly accurate and predictive low-dimensional modeling from data for a number of non-linearizable phenomena. Our construct relies on the recent theory of spectral submanifolds (SSMs), the smoothest invariant manifolds that act as nonlinear continuations of non-resonant eigenspaces from the linearization of a system at a stationary state (fixed point, periodic orbit or quasiperiodic orbit^29^). Using appropriate SSM embeddings^30–32^ and an extended form of the classic normal form theory^33^, we obtain sparse dynamical systems describing the reduced dynamics on the slowest SSMs of the system, which are normally hyperbolic and hence robust under perturbations^34^.

We construct the extended normal form within the slowest SSM as if the eigenvalues of the linearized dynamics within the SSM had zero real parts, although that is not the case. As a result, our normalization procedure will not render the simplest possible (linear) normal form for the SSM dynamics, valid only near the underlying isolated stationary state. Instead, our procedure yields a sparsified nonlinear, polynomial normal form on a larger domain of the SSM that can also capture nearby coexisting stationary states. This fully data-driven normalization algorithm learns the normal form transformation and the coefficients of the normal form simultaneously by minimizing an appropriately defined conjugacy error between the unnormalized and normalized SSM dynamics.

For a generic observable of an oscillatory dynamical system without an internal resonance, a two-dimensional data-driven model calculated on the slowest SSM of the system turns out to capture the correct asymptotic dynamics. Such an SSM-reduced model is valid on domains in which the nonlinearity and any possible external forcing are strong enough to create nonlinearizable dynamics, yet are still moderate enough to render the eigenspace of the linear system relevant. More generally, oscillatory systems with *m* independent internal resonances in their spectrum can be described by reduced models on $2\left(m+1\right)$-dimensional SSMs. In both the resonant and the nonresonant cases, the models can be refined by increasing the degree of their nonlinearity rather than by increasing their dimension. As we show in examples, the resulting SSM-based models are explicit, deterministic and even have the potential to predict system behavior outside the range of the training data away from bifurcations. Most importantly, we find that the models also accurately predict forced response, even though they are only trained on data collected from unforced systems.

We illustrate the power of data-driven SSM-reduced models on high-dimensional numerically generated data sets and on experimental data. These and further examples are also available as MATLAB^®^ live scripts, which are part of a general open-source package, SSMLearn, that performs this type of model reduction and prediction for arbitrary data sets.

## Results

### Spectral submanifolds and their reduced dynamics

A recent result in dynamical systems is that all eigenspaces (or spectral subspaces) of linearized systems admit unique nonlinear continuations under well-defined mathematical conditions. Specifically, spectral submanifolds (SSMs), as defined by^29^, are the unique smoothest invariant manifolds that serve as nonlinear extensions of spectral subspaces under the addition of nonlinearities to a linear system. The SSM formulation and terminology we use here is due to^29^; the Methods section “Existence of SSMs” discusses the history of these results and further technical details.

We consider *n*-dimensional dynamical systems of the form

1 $$\dot{{{{{{{{\bf{x}}}}}}}}}={{{{{{{\bf{A}}}}}}}}{{{{{{{\bf{x}}}}}}}}+{{{{{{{{\bf{f}}}}}}}}}_{0}({{{{{{{\bf{x}}}}}}}})+\epsilon {{{{{{{{\bf{f}}}}}}}}}_{1}({{{{{{{\bf{x}}}}}}}},{{{{{{{\boldsymbol{\Omega }}}}}}}}t;\epsilon ),\qquad {{{{{{{{\bf{f}}}}}}}}}_{0}({{{{{{{\bf{x}}}}}}}})={{{{{{{\mathcal{O}}}}}}}}({\left\vert{{{{{{{\bf{x}}}}}}}}\right\vert}^{2}),\qquad 0\le \epsilon \ll 1,$$

with a constant matrix ${{{{{{{\bf{A}}}}}}}}\in {{\mathbb{R}}}^{n\times n},$ and with class *C*^*r*^ functions ${{{{{{{{\bf{f}}}}}}}}}_{0}:{{{{{{{\mathcal{U}}}}}}}}\to {{\mathbb{R}}}^{n}$ and ${{{{{{{{\bf{f}}}}}}}}}_{1}:{{{{{{{\mathcal{U}}}}}}}}\times {{\mathbb{T}}}^{\ell }\to {{\mathbb{R}}}^{n}$, where ${{\mathbb{T}}}^{\ell }={S}^{1}\times \ldots \times {S}^{1}$ is the *ℓ*-dimensional torus. The elements of the frequency vector ${{{{{{{\boldsymbol{\Omega }}}}}}}}{\mathbb{\in }}{{\mathbb{R}}}^{\ell }$ are rationally independent, and hence the function **f**_1_ is quasiperiodic in time. The assumed degree of smoothness for the right-hand side of (1) is $r\in {{\mathbb{N}}}^{+}\cup \left\{\infty ,a\right\}$, with *a* referring to analytic. The small parameter *ϵ* signals that the forcing in system (1) is moderate so that the structure of the autonomous part is still relevant for the full system dynamics. Rigorous mathematical results on SSMs are proven for small enough *ϵ*, but continue to hold in practice for larger values of *ϵ* as well, as we will see in examples. Note that eq. (1) describes equations of motions of physical oscillatory systems. It does not cover phenomenological models of phase oscillators, such as the Kuramoto model^35^.

The eigenvalues ${\lambda }_{j}={\alpha }_{j}+{{{{{{{\rm{i}}}}}}}}{\omega }_{j}\in {\mathbb{C}}$ of **A**, with multiplicities counted, are ordered based on their real parts, ${{{{{{{\rm{Re}}}}}}}}{\lambda }_{j}$, as

2 $${{{{{{{\rm{Re}}}}}}}}{\lambda }_{n}\le {{{{{{{\rm{Re}}}}}}}}{\lambda }_{n-1}\le \ldots \ldots \le {{{{{{{\rm{Re}}}}}}}}{\lambda }_{1}.$$

Their corresponding real *modal subspaces* (or eigenspaces), ${E}_{j}\subset {{\mathbb{R}}}^{n}$, are spanned by the imaginary and real parts of the corresponding eigenvectors and generalized eigenvectors of **A**. To analyze typical systems, we assume that ${{{{{{{\rm{Re}}}}}}}}{\lambda }_{j}={\alpha }_{j}\,\ne\, 0$ holds for all eigenvalues, i.e., ***x*** = **0** is a hyperbolic fixed point for ϵ = 0.

**Fig. 2:**
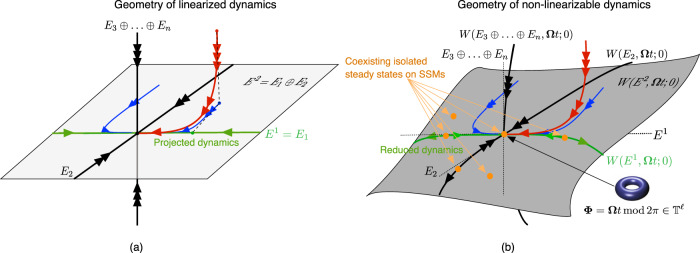
Linear vs. nonlinear model reduction. **a** Reduction of linear dynamics via Galerkin projection. The slowest spectral subspace, *E*^1^ = *E*_1_ (green), and the modal subspace, *E*_2_ (black), span together the second slowest spectral subspace, *E*^2^ = *E*_1_ ⊕ *E*_2_. The full dynamics (red curve) can be projected onto *E*^1^ to yield a reduced slow model without transients. Projection of the full dynamics onto *E*^2^ (blue curve) yields a reduced model that also captures the slowest decaying transient. Further, faster-decaying transients can be captured by projections onto slow spectral subspaces, *E*^*k*^, with *k* > 1. **b** Reduction of nonlinearizable dynamics via restriction to spectral submanifolds (SSMs) in the *ϵ* = 0 limit of nonlinear, non-autonomous systems forced with *ℓ* frequencies. An SSM, *W*(*E*, **Ω***t*; 0), is the unique, smoothest, nonlinear continuation of a nonresonant spectral subspace *E*. Specifically, the slowest SSM, *W*(*E*^*k*^, **Ω***t*; 0) (green), is the unique, smoothest, nonlinear continuation of the slowest spectral subspace, *E*^*k*^. Nonlinearizability of the full dynamics follows if isolated stationary states coexist on at least one of the SSMs. The time-quasiperiodic SSMs for *ϵ* > 0, denoted *W*(*E*, **Ω***t*; *ϵ*), are not shown here but they are ${{{{{{{\mathcal{O}}}}}}}}(\epsilon )$*C*^*r*^-close to the structures shown, as discussed by^29^.

A *spectral subspace* ${E}_{{j}_{1},\ldots ,{j}_{q}}$ is a direct sum

3 $${E}_{{j}_{1},\ldots ,{j}_{q}}={E}_{{j}_{1}}\oplus {E}_{{j}_{2}}\oplus \ldots \oplus {E}_{{j}_{q}}$$

of an arbitrary collection of modal subspaces, which is always an invariant subspace for the linear part of the dynamics in (1). Classic examples of spectral subspaces are the stable and unstable subspaces, comprising all modal subspaces with ${{{{{{{\rm{Re}}}}}}}}{\lambda }_{k} \, < \, 0$ and ${{{{{{{\rm{Re}}}}}}}}{\lambda }_{k} \, > \, 0$, respectively. Projections of the linearized system onto the nested hierarchy of slow spectral subspaces,

4 $${E}^{1}\subset {E}^{2}\subset {E}^{3}\subset \ldots ,\qquad {E}^{k}:= {E}_{1,\ldots ,k},\quad k=1,\ldots ,n,$$

provide exact reduced-order models for the linearized dynamics over an increasing number of time scales under increasing *k*, as sketched in panel (a) of Fig. 2. This is why a Galerkin projection onto *E*^*k*^ is an exact model reduction procedure for linear systems, whose accuracy can be increased by increasing *k*. A fundamental question is whether nonlinear analogues of spectral subspaces continue to organize the dynamics under the addition of nonlinear and time-dependent terms in the full system (1).

Let us fix a specific spectral subspace $E={E}_{{j}_{1},\ldots ,{j}_{q}}$ within either the stable or the unstable subspace. If *E* is non-resonant (i.e., no nonnegative, low-order, integer linear combination of the spectrum of **A**∣_*E*_ is contained in the spectrum of **A** outside *E*), then *E* has infinitely many nonlinear continuations in the system (1) for *ϵ* small enough^29^. These invariant manifolds are of smoothness class *C*^Σ(*E*)^, with the spectral quotient Σ(*E*) measuring the ration of the fastest decay exponent outside *E* to the slowest decay exponent inside *E* (see eq. (13) of the Methods section “Existence of SSMs”). All such manifolds are tangent to *E* for *ϵ* = 0, have the same quasiperiodic time dependence as **f**_1_ does and have a dimension equal to that of *E*.

Of these infinitely may invariant manifolds, however, there will be a unique smoothest one, the *spectral submanifold* (SSM) of *E*, denoted *W*(*E*, **Ω***t*; *ϵ*). This manifold is *C*^*r*^ smooth if *r* > Σ(*E*) and can therefore be approximated more accurately than the other infinitely many nonlinear continuations of *E*. In particular, SSMs have convergent Taylor expansions if the dynamical system (1) is analytic (*r* = *a*). Then the reduced dynamics on a slow SSM, *E*^*k*^, can be approximated with arbitrarily high accuracy using arbitrarily high-order Taylor expansions, without ever increasing the dimension of *E*^*k*^, see panel (b) of Fig. 2. Such an approximation for dynamical systems with known governing equations is now available for any required order of accuracy via the open-source MATLAB^®^ package SSMTool^36^. In contrast, reduced models obtained from projection-based procedures can only be improved by increasing their dimensions.

The nearby coexisting stationary states in Fig. 2 happen to be contained in the SSM. In specific examples, however, these states may also be off the SSM, contained instead in one of the infinitely many additional nonlinear continuations, $\tilde{W}(E,{{{{{{{\boldsymbol{\Omega }}}}}}}}t;\epsilon )$, of the spectral subspace *E*. The Taylor expansion of the dynamics on $\tilde{W}(E,{{{{{{{\boldsymbol{\Omega }}}}}}}}t;\epsilon )$ and *W*(*E*, **Ω***t*; *ϵ*) are, however, identical up to order Σ(*E*). Therefore, the reduced models we will compute on the SSM *W*(*E*, **Ω***t*; *ϵ*) also correctly capture the nearby stationary states on $\tilde{W}(E,{{{{{{{\boldsymbol{\Omega }}}}}}}}t;\epsilon )$, as long as the polynomial order of the model stays below Σ(*E*). In large physical systems, this represents no limitation, given that Σ(*E*) ≫ 1.

### Embedding SSMs via generic observables

If at least some of the real parts of the eigenvalues in (2) are negative, then longer-term trajectory data for system (1) will be close to an attracting SSM, as illustrated in panel (b) of Fig. 2. This is certainly the case for data from experiments that are run until a nontrivial, attracting steady state emerges, see, e.g., in panel (e) of Fig. 1. Measurements of trajectories in the full phase space, however, are seldom available from such experiments. Hence, if data about system (1) is only available from observables, the construction of SSMs and their reduced dynamics has to be carried out in the space of those observables.

**Fig. 3:**
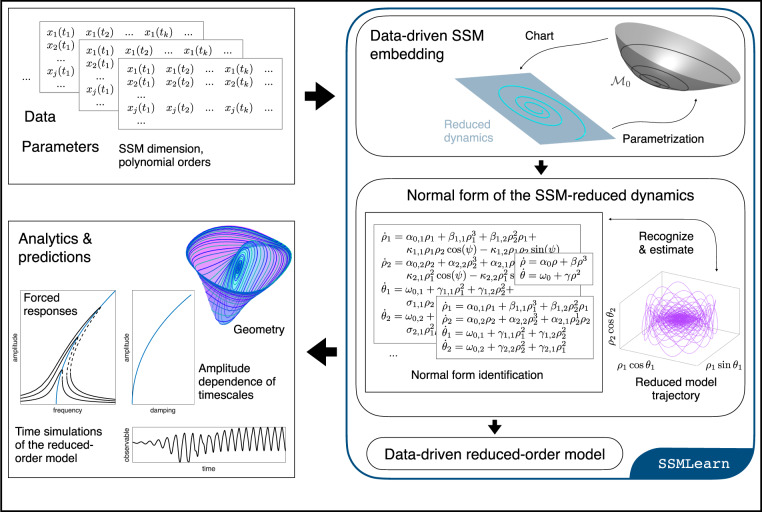
Schematics of SSMLearn. First, he data-driven, SSM-based model reduction algorithm implemented in SSMLearn diagnoses and approximates the dominant SSM from the input data. Next, it constructs a data-driven reduced-order model as an extended normal form on the SSM. Finally, the algorithm uses this model to predict individual unforced trajectories and the response of the system under additional forcing.

An extended version of Whitney’s embedding theorem guarantees that almost all (in the sense of prevalence) smooth observable vectors ${{{{{{{\bf{y}}}}}}}}({{{{{{{\bf{x}}}}}}}})=({y}_{1}({{{{{{{\bf{x}}}}}}}}),,...,{y}_{p}({{{{{{{\bf{x}}}}}}}}))\in {{\mathbb{R}}}^{p}$ provide an embedding of a compact subset ${{{{{{{\mathcal{C}}}}}}}}\subset W(E,{{{{{{{\boldsymbol{\Omega }}}}}}}}t;\epsilon )$ of a *d*-dimensional SSM, *W*(*E*, **Ω***t*; *ϵ*), into the observable space ${{\mathbb{R}}}^{p}$ for high enough *p*. Specifically, if we have *p* > 2(*d* + *ℓ*) simultaneous and independent continuous measurements, **y**(**x**), of the *p* observables, then almost all maps ${{{{{{{\bf{y}}}}}}}}:{{{{{{{\mathcal{C}}}}}}}}\to {{\mathbb{R}}}^{p}$ are embeddings of ${{\mbox{}}}{{{{{{{\mathcal{C}}}}}}}}{{\mbox{}}}$^37^, and hence the top right plot of Fig. 3 is applicable with probability one.

In practice, we may not have access to *p* > 2(*d* + *ℓ*) independent observables and hence cannot invoke Whitney’s theorem. In that case, we invoke the Takens delay embedding theorem^38^, which covers observable vectors built from *p* uniformly sampled, consecutive measured instances of a single observable. More precisely, if *s*(*t*) is a generic scalar quantity measured at times Δ*t* apart, then the observable vector for delay-embedding is formed as ${{{{{{{\bf{y}}}}}}}}(t)=\left(s(t),s(t+{{\Delta }}t),...,s\left(t+(p-1){{\Delta }}t\right)\right)\in {{\mathbb{R}}}^{p}$. We discuss the embedding, ${{{{{{{{\mathcal{M}}}}}}}}}_{0}\subset {{\mathbb{R}}}^{p}$, of an autonomous SSM, *W*(*E*, **Ω***t*_0_; 0), in the observable space ${{\mathbb{R}}}^{p}$ in more detail in the Methods section “Embedding the SSM in the observable space”.

### Data-driven extended normal forms on SSMs

Once the embedded SSM, ${{{{{{{{\mathcal{M}}}}}}}}}_{0}$, is identified in the observable space, we seek to learn the reduced dynamics on ${{{{{{{{\mathcal{M}}}}}}}}}_{0}$. An emerging requirement for learning nonlinear models from data has been model sparsity^4^, without which the learning process would be highly sensitive. The dynamics on ${{{{{{{{\mathcal{M}}}}}}}}}_{0}$, however, is inherently nonsparse, which suggests that we learn its Poincaré normal form^39^ instead. This classic normal form is the simplest polynomial form to which the dynamics can be brought via successive, near-identity polynomial transformations of increasing order.

Near the origin on a slow SSM, however, this simplest polynomial form is just the restriction of the linear part of system (1) to ${{{{{{{{\mathcal{M}}}}}}}}}_{0}$, as long as infinitely many nonresonance conditions are satisfied for the operator **A**^40^. The Poincaré normal form on ${{{{{{{{\mathcal{M}}}}}}}}}_{0}$ would, therefore, only capture the low-amplitude, linearized part of the slow SSM dynamics.

To construct an SSM-reduced model for non-linearizable dynamics, we use *extended normal forms*. This idea is motivated by normal forms used in the study of bifurcations of equilibria on center manifolds depending on parameters^33,41^. In that setting, the normal form transformation is constructed at the bifurcation point where the system is non-linearizable by definition. The same transformation is then used away from bifurcations, even though the normal form of the system would be linear there. One, therefore, gives up the maximal possible simplicity of the normal form but gains a larger domain on which the normal form transformation is invertible and hence captures truly nonlinear dynamics. In our setting, there is no bifurcation at **x** = **0**, but we nevertheless construct our normal form transformation as if the eigenvalues corresponding to the slow subspace *E* were purely imaginary. This procedure leaves additional, near-resonant terms in the SSM-reduced normal form, enhancing the domain on which the transformation is invertible and hence the normal form is valid.

We determine the normal form coefficients directly from data via the minimization of a conjugacy error (see the Methods section). This least-square minimization procedure renders simultaneously the best-fitting normal form coefficients and the best fitting normal form transformation. As we will find in a specific example, this data-driven procedure can yield accurate reduced models even beyond the formal domain of convergence of equation-driven normal forms.

The simplest extended normal form on a slow SSM of an oscillatory system arises when the underlying spectral subspace *E* corresponds to a pair of complex conjugate eigenvalues. Writing in polar coordinates and truncating at cubic order,^42^ finds this normal form on the corresponding two-dimensional, autonomous SSM, ${{{{{{{{\mathcal{M}}}}}}}}}_{0}$, to be

5 $$\begin{array}{ll}\dot{\rho }=\,{\alpha }_{0}\rho +\beta {\rho }^{3},\!\!\!\\ \dot{\theta }=\,{\omega }_{0}+\gamma {\rho }^{2}.\end{array}$$

This equation is also known as the Stuart–Landau equation arising in the unfolding of a Hopf bifurcation^43–45^.

The dynamics of (5) is characteristically nonlinearizable when *α*_0_*β* < 0, given that a limit cycle coexists with the *ρ* = 0 fixed point in that case. Further coexisting steady states will arise when forcing is added to the system, as we discuss in the next section. We note that the cubic normal form on two-dimensional SSMs has also been approximated from data in^46^. That non-sparse procedure fits the full observer dynamics to a low-dimensional, discrete polynomial dynamical system, then performs an analytic SSM reduction and a classic normal form transformation on the SSM.

For higher accuracy, the extended normal form on an oscillatory SSM of dimension 2*m* is of the form

6 $$\begin{array}{c}{\dot{\rho }}_{j}={\alpha }_{j}({{{{{{{\boldsymbol{\rho }}}}}}}},{{{{{{{\boldsymbol{\theta }}}}}}}}){\rho }_{j},\\ \!\!\!\!\!{\dot{\theta }}_{j}={\omega }_{j}({{{{{{{\boldsymbol{\rho }}}}}}}},{{{{{{{\boldsymbol{\theta }}}}}}}}),\end{array}\,\,\,\,\,\,\,j=1,2,...m,\,\,\,\,\,\,{{{{{{{\boldsymbol{\rho }}}}}}}}\in {{\mathbb{R}}}_{+}^{m},\,\,\,\,\,\,{{{{{{{\boldsymbol{\theta }}}}}}}}\in {{\mathbb{T}}}^{m}.$$

If the linearized frequencies are nonresonant, then the functions *α*_*j*_ and *ω*_*j*_ only depend on ***ρ***^42^. Our numerical procedure determines these functions up to the necessary order that ensures a required accuracy for the reduced-order model on the SSM. This is illustrated schematically for a four-dimensional slow SSM (*m* = 2) in the bottom right plot of Fig. 3.

### Predicting forced dynamics from unforced data

With the normalized reduced dynamics (6) on the embedded SSM, ${{{{{{{{\mathcal{M}}}}}}}}}_{0}$, at hand, we can also make predictions for the dynamics of the embedded quasiperiodic SSM, ${{{{{{{{\mathcal{M}}}}}}}}}_{\epsilon }({{{{{{{\boldsymbol{\Omega }}}}}}}}t)$, of the full system (1). This forced SSM is guaranteed to be an ${{{{{{{\mathcal{O}}}}}}}}(\epsilon )$*C*^*r*^-close perturbation of ${{{{{{{{\mathcal{M}}}}}}}}}_{0}$ for moderate external forcing amplitudes. A strict proof of this fact is available for small enough *ϵ* > 0^29^, but as our examples will illustrate, the smooth persistence of the SSM, ${{{{{{{{\mathcal{M}}}}}}}}}_{\epsilon }({{{{{{{\boldsymbol{\Omega }}}}}}}}t)$, generally holds for all moderate *ϵ* values in practice. Such moderate forcing is highly relevant in a number of technological settings, including system identification in structural dynamics and fluid-structure interactions, where the forcing must be moderate to preserve the integrity of the structure.

We discuss the general extended normal form on ${{{{{{{{\mathcal{M}}}}}}}}}_{\epsilon }({{{{{{{\boldsymbol{\Omega }}}}}}}}t)$ in the Methods section “SSM dynamics via extended normal forms”. In the simplest and most frequent special case, the external forcing is periodic (*ℓ* = 1) and ${{{{{{{{\mathcal{M}}}}}}}}}_{\epsilon }({{\Omega }}t)$ is the embedding of the slowest, two-dimensional SSM corresponding to a pair of complex conjugate eigenvalues. Using the modal forcing amplitude *f*_1,1_ and modal phase shift *ϕ*_1,1_ in the general normal form (25)^47^, introduces the new phase coordinate *ψ* = *θ* − Ω*t* − *ϕ*_1,1_ and lets *f* = *f*_1,1_, *α* = *α*_1_, *ω* = *ω*_1_ to obtain the planar, autonomous, extended normal form on ${{{{{{{{\mathcal{M}}}}}}}}}_{\epsilon }({{\Omega }}t)$ as

7 $$\dot{\rho } 	=\alpha (\rho )\rho +f\sin \psi ,\\ \dot{\psi } 	=\omega (\rho )-{{\Omega }}+\frac{f}{\rho }\cos \psi$$

at leading order in *ϵ*. All stable and unstable periodic responses on the SSM are fixed points of system (7), with their amplitudes *ρ*_0_ and phases *ψ*_0_ satisfying the equations

8 $${{\Omega }}=\omega ({\rho }_{0})\pm \sqrt{\frac{{f}^{2}}{{\rho }_{0}^{2}}-{\alpha }^{2}({\rho }_{0})},\quad {\psi }_{0}={\tan }^{-1}\left[\frac{\alpha \left({\rho }_{0}\right)}{\omega \left({\rho }_{0}\right)-{{\Omega }}}\right].$$

The first analytic formula in (8) predicts the *forced response curve* (FRC) of system (1), i.e., the relationship between response amplitude, forcing amplitude and forcing frequency, from the terms *α*(*ρ*) and *ω*(*ρ*) of the extended normal form of the autonomous SSM, ${{{{{{{{\mathcal{M}}}}}}}}}_{0}$. These terms are constructed from trajectories of the unforced system, thus eq. (8) predicts the behavior of a nonlinearizable dynamical system under forcing based solely on unforced training data. The stability of the predicted periodic response follows from a simple linear analysis at the corresponding fixed point of the ODE (7). The first formula in (8) also contains another frequently used notion of nonlinear vibration analysis, the *dissipative backbone curve ω*(*ρ*), which describes the instantaneous amplitude-frequency relation along freely decaying vibrations within the SSM.

As we will also show in examples, our unforced model-based predictions for forced periodic response (see the Methods section “Prediction of forced response from unforced training data”) are confirmed by numerical simulation or dedicated laboratory experiments on forced systems.

### Examples

We now illustrate data-driven, SSM-based modeling and prediction on several numerical and experimental data sets describing non-linearizable physical systems. Further applications are described in^48^. Both the numerical and the experimental data sets were initialized without knowledge of the exact SSM. All our computations have been carried out by the publicly available MATLAB^®^ package, SSMLearn, whose repository also contains further examples not discussed here. The main algorithm behind SSMLearn is illustrated in Fig. 3, with more detail given in the Methods section “Summary of the algorithm”.

To quantify the errors of an SSM-based reduced model, we use the *normalized mean-trajectory-error* (NMTE). For *P* observations of the observable vector **y**_*j*_ and their model-based reconstructions, ${\hat{{{{{{{{\bf{y}}}}}}}}}}_{j}$, this modeling error is defined as

9 $${{{{{{{\rm{NMTE}}}}}}}}=\frac{1}{\parallel \underline{{{{{{{{\bf{y}}}}}}}}}\parallel }\frac{1}{P}\mathop{\sum }\limits_{j=1}^{P}\parallel {{{{{{{{\bf{y}}}}}}}}}_{j}-{\hat{{{{{{{{\bf{y}}}}}}}}}}_{j}\parallel\!.$$

Here $\underline{{{{{{{{\bf{y}}}}}}}}}$ is a relevant normalization vector, such as the data point with the largest norm. When validating the reduced dynamics for a given testing trajectory, we run the reduced model from the same initial condition for the comparison. Increasing the order of the truncated normal form polynomials in eq. (6) generally reduces the NMTE error to any required level but excessively small errors can lead to overfitting. In our examples, we will be allowing model errors in the order of 1% − 4% to avoid overfitting.

**Fig. 4:**
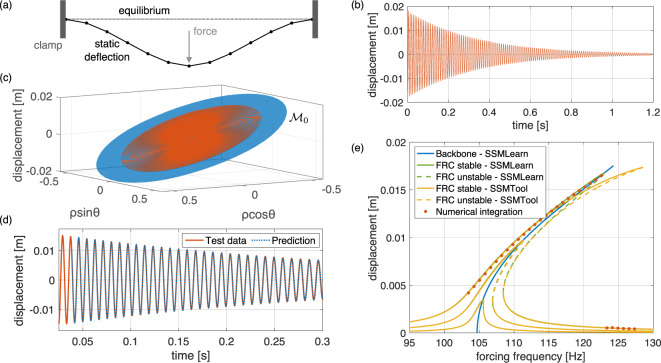
Construction of a data-driven nonlinear reduced-order model on the slowest SSM of a von Kármán beam. (**a**) System setup and the initial condition for the decaying training trajectory shown in (**b**) in terms of the midpoint displacement. (**c**) The SSM, ${{{{{{{{\mathcal{M}}}}}}}}}_{0}$, in the delay embedding space, shown along with the reconstructed test trajectory in extended normal form coordinates. (**d**) Zoom of the prediction of the reduced order model for the test trajectory not used in learning ${{{{{{{{\mathcal{M}}}}}}}}}_{0}$. (**e**) Closed-form backbone curve and forced response curve (FRC) predictions (*ϵ* > 0, *ℓ* = 1) by SSMLearn are compared with analytic FRC calculations performed by SSMTool^36^ and with results from numerical integration of the forced-damped beam.

As a first example, we consider a finite-element discretization of a von Kármán beam with clamped-clamped boundary conditions^49^, shown in panel (a) of Fig. 4. In contrast to the classic Euler-Bernoulli beam, the von Kármán model captures moderate deformations by including a nonlinear, quadratic term in the kinematics. We first construct a 33 degree-of-freedom, damped, unforced finite element model (i.e., *n* = 66 and *ϵ* = 0 in eq. (1)) for an aluminum beam of length 1 [m], width 5 [cm], thickness 2 [cm] and material damping modulus 10^6^ [] (see the Supplementary Information for more detail).

Our objective is to learn from numerically generated trajectory data the reduced dynamics on the slowest, two-dimensional SSM, *W*(*E*_1_), of the system, defined over the slowest two-dimensional (*d* = 2) eigenspace *E*_1_ of the linear part. To do so, we generate two trajectories starting from initial beam deflections caused by static loading of 12 [kN] and 14 [kN] at the midpoint, as shown in panel (a) of Fig. 4. The latter trajectory, shown in panel (b) of Fig. 4, is used for training, the other for testing. Along the trajectories, we select our single observable *s*(*t*) to be the midpoint displacement of the beam.

The beam equations are analytic (*r* = *a*), and hence the SSM, *W*(*E*_1_), admits a convergent Taylor expansion near the origin. The minimal embedding dimension for the two-dimensional, *W*(*E*_1_), as required by Whitney’s theorem, is *p* = 5, which is not satisfied by our single scalar observable *s*(*t*). We therefore employ delay-embedding using ${{{{{{{\bf{y}}}}}}}}(t)=\left(s(t),s(t+{{\Delta }}t),\ldots ,s(t+4{{\Delta }}t)\right)$ with Δ*t* = 0.0955 [ms]. By Takens’s theorem, this delayed observable embeds the SSM in ${{\mathbb{R}}}^{5}$ with probability one.

A projection of the embedded SSM, ${{{{{{{{\mathcal{M}}}}}}}}}_{0}\in {{\mathbb{R}}}^{5},$ onto three coordinates is shown in panel (c) of Fig. 4. On ${{{{{{{{\mathcal{M}}}}}}}}}_{0}$, SSMLearn returns the 7th-order extended normal form

10 $$\dot{\rho } 	=\alpha (\rho )\rho ,\quad \alpha (\rho )=-3.02-5.79{\rho }^{2}+57.5{\rho }^{4}-191{\rho }^{6},\\ \dot{\theta } 	=\omega (\rho ),\,\,\quad \omega (\rho )=658+577{\rho }^{2}-347{\rho }^{4}-387{\rho }^{6},$$

to achieve our preset reconstruction error bar of 3% on the test trajectory (NMTE = 0.027), shown in panel (d) of Fig. 4.

We now use the model (10), trained on a single decaying trajectory, to predict the forced response of the beam for various forcing amplitudes and frequencies in closed form. We will then compare these predictions with analytic forced response computations for the forced SSM, ${{{{{{{{\mathcal{M}}}}}}}}}_{\epsilon }({{\Omega }}t)$, obtained from SSMTool^36^ and with numerical simulations of the damped-forced beam. The periodic forcing is applied at the midpoint node; the Taylor expansion order in SSMTool for the analytically computed dynamics on ${{{{{{{{\mathcal{M}}}}}}}}}_{\epsilon }({{\Omega }}t)$ is set to 7, as in (10). Panel (e) of Fig. 4 shows the FRCs (green) and the backbone curve (blue) predicted by SSMLearn based on formula (8) from the single unforced trajectory in panel (b) of Fig. 4. To obtain the relevant forcing amplitudes *f* in the delay-observable space, we have followed the calibration procedure described in the Methods section “Prediction of forced response from unforced training data” for the forcing values $\left|\epsilon {{{{{{{{\bf{f}}}}}}}}}_{1}\right|=15,45,95$ [N] at the single forcing frequency Ω = 103.5 [Hz]. Recall that coexisting stable (solid lines) and unstable (dashed lines) periodic orbits along the same FRC are hallmarks of non-linearizable dynamics and hence cannot be captured by the model reduction techniques we reviewed in the Introduction for linearizable systems.

The data-based prediction for the FRCs agrees with the analytic FRCs for low forcing amplitudes but departs from it for higher amplitudes. Remarkably, as the numerical simulations (red) confirm, the data-based FRC is the correct one. The discrepancy between the two FRCs for large amplitudes only starts decreasing under substantially higher-order Taylor series approximations used in SSMTool (see the Supplementary Information). This suggests the use of the data-based approach for this class of problems even if the exact equations of motion are available.

**Fig. 5:**
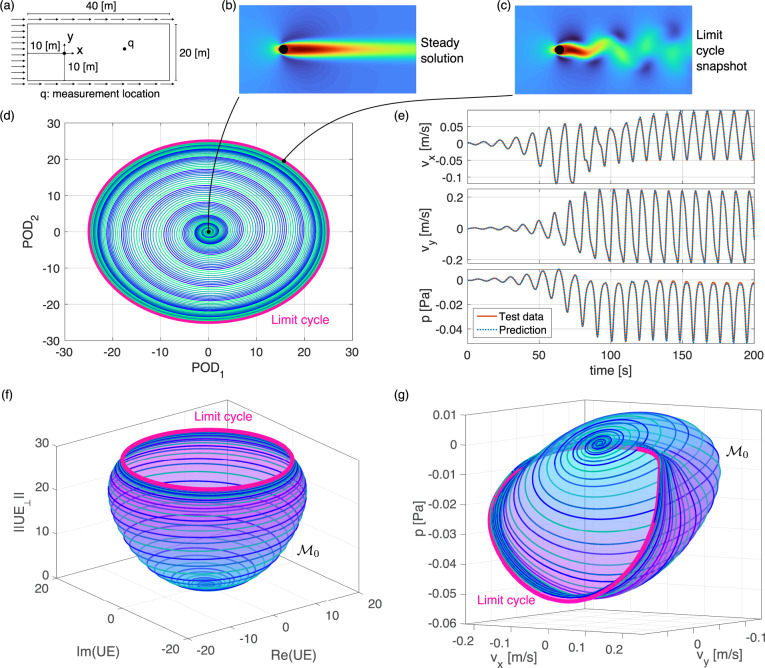
Data-driven nonlinear SSM-reduced model on the unstable manifold of the steady solution of the flow past a cylinder. **a** Problem setup. **b**, **c** Snapshots of the steady solution and the time-periodic vortex-shedding solution (limit cycle, in magenta). **d** Trajectories projected on the 2-dim. subspace spanned by the two-leading POD modes of the limit cycle. **e** Model-based reconstruction of the test trajectory (not used in learning the SSM) in terms of velocities and pressures measured at a location *q* shown in plot **a**. **f** The SSM formed by the unstable manifold of the origin, along with some reduced trajectories, plotted over the unstable eigenspace *UE* ≡ *E*_1_; ∥UE_⊥_∥ denotes the normed projection onto the orthogonal complement UE_⊥_. **g** Same but projected over velocity and pressure coordinates.

As a second example, we consider the classic problem of vortex shedding behind a cylinder^8^. Our input data for SSM-based reduced modeling are the velocity and pressure fields over a planar, open fluid domain with a hole representing the cylinder section, as shown in panel (a) of Fig. 5. The boundary conditions are no-slip on the circular inner boundary, standard outflow on the outer boundary at the right side, and fixed horizontal velocity on the three remaining sides^50^. The Reynolds number for this problem is the ratio between the cylinder diameter times the inflow velocity and the kinematic viscosity of the fluid.

Available studies^8,50,51^ report that, at low Reynolds number, the two-dimensional unstable manifold, *W*^*u*^(*SS*), of the wake-type steady solution, *SS*, in panel (b) of Fig. 5 connects *SS* to the limit cycle shown in panel (c) of Fig. 5. Here we evaluate the performance of SSMLearn on learning this unstable manifold as an SSM, along with its reduced dynamics, from trajectory data at Reynolds number equal to 70. For this SSM, we again have *d* = 2 and *r* = *a*, as in our previous example. There is no external forcing in this problem, and hence we have *ϵ* = 0 in eq. (1). In contrast to prior studies that often consider a limited number of observables^8,51,52^, here we select the full phase space of the discretized Navier-Stokes simulation to be the observable space for illustration, which yields *n* = *p* = 76, 876 in eq. (1). We generate nine trajectories numerically, eight of which will be used for training and one for testing the SSM-based model.

The nine initial conditions of our input trajectory data are small perturbations from the wake-type steady solution along its unstable directions, equally spaced on a small amplitude circle on this unstable plane. All nine trajectories quickly converge to the unstable manifold and then to the limit cycle representing periodic vortex shedding.

We choose to parametrize the SSM, ${{{{{{{{\mathcal{M}}}}}}}}}_{0}={W}^{u}(SS)$, with two leading POD modes of the limit cycle, which have been used in earlier studies for this problem. The training trajectories projected onto these two POD modes are shown in panel (d) of Fig. 5. To limit the modeling error (9) to less than NMTE = 1%, SSMLearn requires a polynomial order of 18 in the SSM computations. For this order, our approach can accommodate the strong mode deformation observed for this problem^51^, manifested by a fold of the SSM over the unstable eigenspace in panel (f) of Fig. 5. Panel (g) of Fig. 5 shows the strongly nonlinear geometry of ${{{{{{{{\mathcal{M}}}}}}}}}_{0}$ projected to the observable subspace formed by the velocities and the pressure of a probe point in the wake.

To capture the SSM-reduced dynamics with acceptable accuracy, we need to compute the extended normal form up to order 11, obtaining

11 $$\begin{array}{c}\dot{\rho }=\alpha (\rho )\rho =0.0584\rho -0.479{\rho }^{3}+1.27{\rho }^{5}+6.80{\rho }^{7}-58.9{\rho }^{9}+108{\rho }^{11},\\ \!\!\!\!\!\!\!\!\!\dot{\theta }=\omega (\rho )\,=0.553+0.441{\rho }^{2}-3.38{\rho }^{4}+55.5{\rho }^{6}-321{\rho }^{8}+626{\rho }^{10}.\end{array}$$

To describe a transition qualitatively from a fixed point to a limit cycle, the reduced-order dynamical model should be at least of cubic order^51^. Capturing the qualitative behavior (i.e., the unstable fixed point and the stable limit cycle), however, does not imply a low NMTE error for the model. Indeed, the data-driven cubic normal form for this example gives a reconstruction error of NMTE = 117% normalized over the limit cycle amplitude, mainly arising from an out-of-phase convergence to the limit cycle along the testing trajectory. In contrast, the ${{{{{{{\mathcal{O}}}}}}}}(11)$ normal form in eq. (11) reduced this error drastically to NMTE = 3.86% on the testing trajectory, as shown in panel (e) of Fig. 5.

We show in Section 1.2.3 of the Supplementary Information that for comparable accuracy, the Sparse Identification of Nonlinear DYnamics (SINDy) approach of^4^ returns non-sparse nonlinear models for this example. Similarly, while the DMD^13^ can achieve highly accurate curve-fitting on the available training trajectories with a high-dimensional linear model, that model only captures linearizable dynamics near the origin. As a consequence, its trajectories grow without bound over longer integration times and hence fail to capture the limit cycle.

As a third example, we consider fluid oscillations in a tank, which exhibit highly nonlinear characteristics^53^. To describe such non-linearizable softening effects observed in the sloshing motion of surface waves, Duffing-type models have been proposed^54^. While amplitude variations observed in forced experiments can be fitted to forced softening Duffing equations, nonlinear damping remains a challenge to identify^55^.

**Fig. 6:**
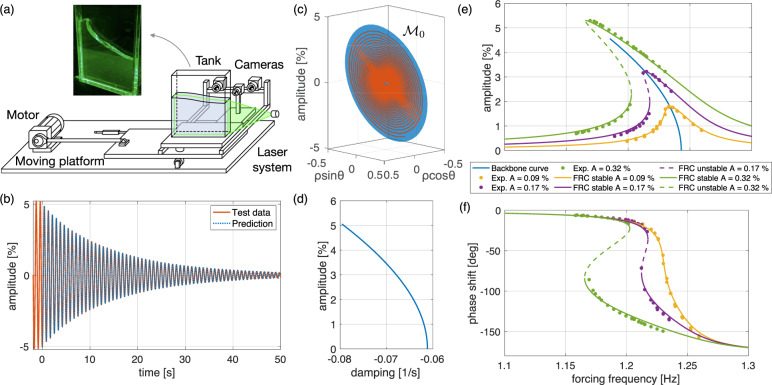
Data-driven nonlinear reduced-order model on the slowest SSM of fluid sloshing in a tank. **a** Setup for the sloshing experiment^55^. **b** Decaying model-testing trajectory and its reconstruction from an unforced, SSM-based model **c** The geometry of the embedded SSM **d** Nonlinear damping *α*(*ρ*) from the SSM-reduced dynamics **e**, **f** Closed form, SSM-based predictions of the FRCs and the response phases *ψ*_0_ for three different forcing amplitudes (solid lines), with their experimental confirmation superimposed (dots).

The experiments we use to construct an SSM-reduced nonlinear model for sloshing were performed in a rectangular tank of width 500 [mm] and depth 50 [mm], partially filled with water up to a height of 400 [mm], as shown in panel (a) of Fig. 6. The tank was mounted on a platform excited harmonically by a motor. The surface level was detected via image processing from a monochrome camera. As an observable *s*(*t*) we used the horizontal position of the computed center of mass of the water at each time instant, normalized by the tank width. This physically meaningful scalar is robust with respect to image evaluation errors^55^.

We identify the unforced nonlinear behavior of the system from data obtained in resonance decay experiments^56^. In those experiments (as in Fig. panel (a) of 6, but with a shaker instead of a motor), once a periodic steady state is reached under periodic horizontal shaking of the tank, the shaker is turned off and the decaying sloshing is recorded. We show such a decaying observable trajectory (orange line) in panel (b) of Fig. 6, with the shaker switched off slightly before zero time. This damped oscillation is close, by construction, to the two-dimensional, slowest SSM of the system. We use three such decaying observer trajectories (two for training and one for model testing) for the construction of a two-dimensional (*d* = 2), autonomous, SSM-based reduced-order model for *s*(*t*). For delay embedding dimension, we again pick *p* = 5, the minimal value guaranteed to be generically correct for embedding the SSM by Takens’s theorem. The delay used in sampling *s*(*t*) is Δ*t* = 0.033 [s]. For this input and for a maximal reconstruction error of 2%, SSMLearn identifies a nearly flat SSM in the delayed observable space–see panel (c) of Fig. 6–with a cubic extended normal form

12 $$\dot{\rho }=-0.063179\rho -0.041214{\rho }^{3},\quad \dot{\theta }=7.8144-1.5506{\rho }^{2}.$$

This lowest-order, Stuart–Landau-type normal form, cf. (5), already constitutes an accurate reduced-order model with NMTE = 1.88% on the testing data set, see panel (b) of Fig. 6. The amplitude-dependent nonlinear damping, *α*(*ρ*), provided by this model is plotted in panel (d) of Fig. 6 with respect to the physical amplitude.

In another set of experiments with the setup of panel (a) Fig. 6, steady states of periodically forced sloshing were measured in sweeps over a range of forcing frequencies under three different shaker amplitudes. As in the previous beam example, we identify the corresponding forcing amplitude, *f*, in (7) at the maximal amplitude response of each frequency sweep. Shown in panels (e, f) of Fig. 6, the closed-form predictions for FRCs from eq. (8) (solid lines) match closely the experimental FRCs (dots). Given the strong nonlinearity of the FRC, any prediction of this curve from a DMD-based model is bound to be vastly inaccurate, as we indeed show in Section 1.3 of the Supplementary information.

The phase *ψ*_0_ of the forced response relative to the forcing has been found difficult to fit to forced Duffing-type models^55^, but the present modeling methodology also predicts this phase accurately using the second expression in (8). The blue curve in panel (e) of Fig. 6 shows the backbone curve of decaying vibrations, which terminates at the highest amplitude occurring in the training data set. This plot therefore shows that the closed-form FRC predictions obtained from the SSM-based reduced model are also effective for response amplitudes outside the training range of the reduced model.

## Discussion

We have described a data-driven model reduction procedure for non-linearizable dynamical systems with coexisting isolated stationary states. Our approach is based on the recent theory of spectral submanifolds (SSMs), which are the smoothest nonlinear continuations of spectral subspaces of the linearized dynamics. Slow SSMs form a nested hierarchy of attractors and hence the dynamics on them provide a hierarchy of reduced-order models with which generic trajectories synchronize exponentially fast. These SSMs and their reduced models smoothly persist under moderate external forcing, yielding low-dimensional, mathematically exact reduced-order models for forced versions of the same dynamical system. The normal hyperbolicity of SSMs also ensures their robustness under small noise.

All these results have been implemented in the open-source MATLAB^®^ package, SSMLearn, which we have illustrated on data sets arising from forced nonlinear beam oscillations, vortex shedding behind a cylinder and water sloshing in a vibrating tank. For all three examples, we have found that two-dimensional data-driven extended normal forms on the slowest SSMs provide sparse yet accurate models of non-linearizable dynamics in the space of the chosen observables. Beyond matching training and testing data, SSM-reduced models prove their intrinsic, qualitative meaning by predicting non-linearizable, forced steady states purely from decaying, unforced data.

In this brief report, examples of higher-dimensional SSMs and multi-harmonic forcing have not been considered, even though SSMLearn is equipped to handle them. Higher-dimensional SSMs are required in the presence of internal resonances or in non-resonant problems in which initial transients also need to be captured more accurately. A limitation of our approach for non-autonomous systems is the assumption of quasiperiodic external forcing. Note, however, that even specific realizations of stochastic forcing signals can be approximated arbitrarily closely with quasiperiodic functions over any finite time interval of interest. A further limitation in our work is the assumption of smooth system dynamics. For data from non-smooth systems, SSMLearn will nevertheless return an equivalent smooth reduced-order model whose accuracy is a priori known from the available mean-squared error of the SSM fitting and conjugacy error of the normal form construction. We are addressing these challenges in ongoing work to be reported elsewhere. Further applications of SSMLearn to physical problems including higher-dimensional coexisting steady states (see, e.g.,^57^) are also underway.

## Methods

### Existence of SSMs

In the context of rigid body dynamics, invariant manifolds providing generalizations of invariant spectral subspaces to nonlinear systems were first envisioned and formally constructed as nonlinear normal modes by^58^ (see^59^ for a recent review of related work). Later studies, however, pointed out the nonuniqueness of nonlinear normal modes in specific examples (^60,61^).

In the mathematics literature,^62^ obtained general results on the existence, smoothness and degree of uniqueness of such invariant manifolds for mappings on Banach spaces. These results use a special parameterization method to construct the manifolds even in evolutionary partial differential equations that admit a well-posed flow map in both time directions (see^63^ for a mechanics application). The results have been extended to a form applicable to dynamical systems with quasiperiodic time dependence^64^. An extensive account of the numerical implementation of the parametrization method with a focus on computing invariant tori and their whiskers in Hamiltonian systems is also available^65,29^ Discussed the existence of the SSM, *W*(*E*, **Ω***t*; *ϵ*), depending on its absolute spectral quotient,

13 $${{\Sigma }}(E)={{{{{{{\rm{Int}}}}}}}}\,\left[\frac{\mathop{\max }\limits_{\lambda \in {{{{{{{\rm{Spect}}}}}}}}({{{{{{{\bf{A}}}}}}}}{| }_{S})}| {{{{{{{\rm{Re}}}}}}}}\lambda | }{\mathop{\min }\limits_{{\lambda }_{e}\in {{{{{{{\rm{Spect}}}}}}}}({{{{{{{\bf{A}}}}}}}}{| }_{E})}| {{{{{{{\rm{Re}}}}}}}}{\lambda }_{e}| }\right],$$

where Spect(**A**∣_*S*_) is the stable (unstable) spectrum of **A** if the SSM is stable (unstable). For a stable SSM, Σ(*E*) is the integer part of the quotient of the minimal real part in the spectrum of **A** and the maximal real part of the spectrum of **A** restricted to *E*.

Based on Σ(*E*), we call a *d*-dimensional spectral subspace *E non-resonant* if for any set $\left({m}_{1},\ldots ,{m}_{d}\right)$ of nonnegative integers satisfying $2\le \mathop{\sum }\nolimits_{j = 1}^{d}{m}_{j}\le {{\Sigma }}(E)$, the eigenvalues, *λ*_*k*_, of **A** satisfy

14 $$\mathop{\sum }\limits_{j=1}^{d}{m}_{j}{{{{{{{\rm{Re}}}}}}}}{\lambda }_{j}\,\ne \,{{{{{{{\rm{Re}}}}}}}}{\lambda }_{k},\quad {\lambda }_{k}\in {{{{{{{\rm{Spect}}}}}}}}({{{{{{{\bf{A}}}}}}}})-{{{{{{{\rm{Spect}}}}}}}}({{{{{{{\bf{A}}}}}}}}{| }_{E}).$$

This condition only needs to be verified for resonance orders between 2 and Σ(*E*)^64^. In particular, a 1: 1 resonance between *E*_1_ and *E*_2_ is allowed if $\dim {E}_{1}=\dim {E}_{2}=1$, in which case each strongly resonant spectral subspace gives rise to a unique nearby spectral submanifold.

If *E* violates the nonresonance condition (14), then *E* can be enlarged to a higher-dimensional spectral subspace until the nonresonance relationship (14) is satisfied. In the absence of external forcing (*ϵ* = 0), the nonresonance condition (14) can also be relaxed with the help of the *relative spectral quotient*,

15 $$\sigma (E)={{{{{{{\rm{Int}}}}}}}}\,\left[\frac{\mathop{\max }\limits_{\lambda \in {{{{{{{\rm{Spect}}}}}}}}({{{{{{{\bf{A}}}}}}}}{| }_{S})-{{{{{{{\rm{Spect}}}}}}}}({{{{{{{\bf{A}}}}}}}}{| }_{E})}| {{{{{{{\rm{Re}}}}}}}}\lambda | }{\mathop{\min }\limits_{{\lambda }_{e}\in {{{{{{{\rm{Spect}}}}}}}}({{{{{{{\bf{A}}}}}}}}{| }_{E})}| {{{{{{{\rm{Re}}}}}}}}{\lambda }_{e}| }\right],$$

to the form

16 $$\mathop{\sum }\limits_{j=1}^{d}{m}_{j}{\lambda }_{j}\,\ne\, {\lambda }_{k},\quad {\lambda }_{k}\in {{{{{{{\rm{Spect}}}}}}}}({{{{{{{\bf{A}}}}}}}})-{{{{{{{\rm{Spect}}}}}}}}({{{{{{{\bf{A}}}}}}}}{| }_{E}),\qquad 2\le \mathop{\sum }\limits_{j=1}^{d}{m}_{j}\le \sigma (E).$$

This is indeed a relaxation because condition (16) is only violated if both the real and the imaginary parts of eigenvalues involved are in the exact same resonance with each other. In contrast, (14) is already violated when the real parts are in resonance with each other.

If ${{{{{{{\rm{Re}}}}}}}}{\lambda }_{1} < 0$ in eq. (2) and all *E*^*k*^ subspaces are nonresonant, then the nested set of slow spectral submanifolds,

$$W({E}^{1},{{{{{{{\boldsymbol{\Omega }}}}}}}}t;\epsilon )\subset W({E}^{2},{{{{{{{\boldsymbol{\Omega }}}}}}}}t;\epsilon )\subset W({E}^{3},{{{{{{{\boldsymbol{\Omega }}}}}}}}t;\epsilon )\subset \ldots ,$$

gives a hierarchy of local attractors. All solutions in a vicinity of **x** = **0** approach the reduced dynamics on one of these attractors exponentially fast, as sketched in panel (b) of Fig. 2 for the *ϵ* = 0 limit. As we will see, non-linearizable dynamics tend to emerge on *W*(*E*^*k*^, **Ω***t*; *ϵ*) due to near-resonance between the linearized frequencies within *E*^*k*^ and the forcing frequencies **Ω**. The specific location of nontrivial steady states in *W*(*E*^*k*^, **Ω***t*; *ϵ*) is then determined by a balance between the nonlinearities, damping and forcing.

A resonant *E*^*k*^ subspace can be enlarged by adding the next $k^{\prime}$ modal subspaces to it until ${E}^{k+k^{\prime} }$ in the hierarchy (4) becomes non-resonant and hence admits an SSM, $W({E}^{k+k^{\prime} },{{{{{{{\boldsymbol{\Omega }}}}}}}}t;\epsilon )$. This technical enlargement is also in agreement with the physical expectation that all interacting modes have to be included in an accurate reduced-order model. Finally, we note that SSMs are robust features of dynamical systems: they inherit smooth dependence of the vector field in (1) on parameters^29^.

For discrete-time dynamical systems of the form

17 $${{{{{{{{\bf{x}}}}}}}}}_{k+1}=\tilde{{{{{{{{\bf{A}}}}}}}}}{{{{{{{{\bf{x}}}}}}}}}_{k}+{\tilde{{{{{{{{\bf{f}}}}}}}}}}_{0}({{{{{{{{\bf{x}}}}}}}}}_{k})+\epsilon {\tilde{{{{{{{{\bf{f}}}}}}}}}}_{1}({{{{{{{{\bf{x}}}}}}}}}_{k},{{{{{{{{\boldsymbol{\phi }}}}}}}}}_{k};\epsilon ),\qquad {{{{{{{{\boldsymbol{\phi }}}}}}}}}_{k+1}={{{{{{{{\boldsymbol{\phi }}}}}}}}}_{k}+\tilde{{{{{{{{\boldsymbol{\Omega }}}}}}}}},$$

the above results on SSMs apply based on the eigenvalues *μ*_*k*_ of $\tilde{{{{{{{{\bf{A}}}}}}}}}$. One simply needs to replace *λ*_*k*_ with $\log {\mu }_{k}$ and ${{{{{{{\rm{Re}}}}}}}}{\lambda }_{k}$ with $\log | {\mu }_{k}|$ in formulas (13)-(16)^29^.

We close by noting that in a neighborhood of an SSM, an invariant family of surfaces resembling the role of coordinate planes in a linear system exists^66^. This invariant spectral foliation (ISF) can, in principle, be used to generate a nonlinear analogue of linear modal superposition in a vicinity of a fixed point. Constructing the ISF from data has shown both initial promise and challenges to be addressed.

### Embedding the SSM in the observable space

Originally conceived for autonomous systems, the Takens delay embedding theorem^38^ has been strengthened and generalized to externally forced dynamics^32^. By these results, the embedding for a *d*-dimensional compact SSM subset, ${{{{{{{\mathcal{C}}}}}}}}\subset W(E,{{{{{{{\boldsymbol{\Omega }}}}}}}}t;\epsilon )$, in the delay observable space as ${{{{{{{\mathcal{M}}}}}}}}({{{{{{{\boldsymbol{\Omega }}}}}}}}t)$ is guaranteed for almost all choices of the observable *s*(*t*) if *p* > 2(*d* + *l*) and some generic assumptions regarding periodic motions on ${{{{{{{\mathcal{M}}}}}}}}({{{{{{{\boldsymbol{\Omega }}}}}}}}t)$ are satisfied^37^.

Of highest importance in technological applications is the case of time-periodic forcing (*ℓ* = 1), with frequency ${{{{{{{\boldsymbol{\Omega }}}}}}}}={{\Omega }}\in {\mathbb{R}}$ and period *T* = 2*π*/Ω. In this case, the Whitney and Takens embedding theorems can be applied to the associated period-*T* sampling map (or Poincaré map) ${{{{{{{{\bf{P}}}}}}}}}_{{t}_{0}}:{{\mathbb{R}}}^{n}\to {{\mathbb{R}}}^{n}$ of the system based at time *t*_0_. This map is autonomous and has a time-independent SSM that coincides with the *d*-dimensional SSM, ${{{{{{{\mathcal{M}}}}}}}}({{\Omega }}{t}_{0})$, of the full system (1). In this case, by direct application of the embedding theorems to the discrete dynamical system generated by ${{{{{{{{\bf{P}}}}}}}}}_{{t}_{0}}$, the typically sufficient embedding dimension estimate is improved to *p* > 2*d* for Whitney’s and Takens’s theorem.

Technically speaking, the available data will never be exactly on an SSM, as these embedding theorems assume. By the smoothness of the embeddings, however, points close enough to the SSM in the phase space will be close to ${{{{{{{\mathcal{M}}}}}}}}({{{{{{{\boldsymbol{\Omega }}}}}}}}t)$ in the observable space under the embeddings. Moreover, as slow SSMs attract nearby trajectories exponentially, the distance of observable data from the embedded slow SSM will shrink exponentially fast. Therefore, even under uncorrelated noise in the measurements, mean-squared estimators are suitable for learning slow SSMs from data in the observable space, as we illustrate in the Supplementary Information.

After a possible coordinate shift, the trivial fixed point of the autonomous limit of system (1) will be mapped into the **y** = **0** origin of the observable space. To find an embedded, *d*-dimensional SSM, ${{{{{{{{\mathcal{M}}}}}}}}}_{0}\in {{\mathbb{R}}}^{p}$, attached to this origin for *ϵ* = 0, we focus on observable domains in which ${{{{{{{{\mathcal{M}}}}}}}}}_{0}$ is a graph over its tangent space ${T}_{{{{{{{{\bf{0}}}}}}}}}{{{{{{{{\mathcal{M}}}}}}}}}_{0}$ at the origin **y** = **0**. Such domains always exist and are generally large enough to capture non-linearizable dynamics in most applications (but see below). Note that ${T}_{{{{{{{{\bf{0}}}}}}}}}{{{{{{{{\mathcal{M}}}}}}}}}_{0}$ coincides with the image of the spectral subspace *E* in the observable space.

To learn such a graph-style parametrization for ${{{{{{{{\mathcal{M}}}}}}}}}_{0}$ from data, we define a matrix ${{{{{{{{\bf{U}}}}}}}}}_{1}\in {{\mathbb{R}}}^{n\times d}$ with columns that are orthonormal vectors spanning the yet unknown ${T}_{{{{{{{{\bf{0}}}}}}}}}{{{{{{{{\mathcal{M}}}}}}}}}_{0}$. The reduced coordinates ${{{{{{{\boldsymbol{\eta }}}}}}}}\in {{\mathbb{R}}}^{d}$ for a point ${{{{{{{\bf{y}}}}}}}}\in {{{{{{{{\mathcal{M}}}}}}}}}_{0}$ are then defined as the orthogonal projection ${{{{{{{\boldsymbol{\eta }}}}}}}}={{{{{{{{\bf{U}}}}}}}}}_{1}^{T}{{{{{{{\bf{y}}}}}}}}$. We week a Taylor-expansion for ${{{{{{{{\mathcal{M}}}}}}}}}_{0}$ near the ***η*** = **0** origin, denoting by ***η***^2:*M*^ the family of all monomials of *d* variables from degree 2 to *M*. For example, if *d* = 2 and *M* = 3, then ${{{{{{{{\boldsymbol{\eta }}}}}}}}}^{2:3}={({\eta }_{1}^{2},{\eta }_{1}{\eta }_{2},{\eta }_{2}^{2},{\eta }_{1}^{3},{\eta }_{1}^{2}{\eta }_{2},{\eta }_{1}{\eta }_{2}^{2},{\eta }_{2}^{3})}^{T}$. As a graph over ${T}_{{{{{{{{\bf{0}}}}}}}}}{{{{{{{{\mathcal{M}}}}}}}}}_{0}$, the manifold ${{{{{{{{\mathcal{M}}}}}}}}}_{0}$ is approximated as **y** = **V**_1_***η*** + **V*η***^2:*M*^, where the matrices **V**_1_ and **V** contain coefficients for the *d*-variate linear and nonlinear monomials, respectively. Learning ${{{{{{{{\mathcal{M}}}}}}}}}_{0}$ from a data set of *P* observations **y**_1_, …, **y**_*P*_ then amounts to finding the $({{{{{{{{\bf{U}}}}}}}}}_{1}^{* },{{{{{{{{\bf{V}}}}}}}}}_{1}^{* },{{{{{{{{\bf{V}}}}}}}}}^{* })$ matrices that minimize the mean-square reconstruction error along the training data:

18 $$\begin{array}{ll}({{{{{{{{\bf{U}}}}}}}}}_{1}^{* },{{{{{{{{\bf{V}}}}}}}}}_{1}^{* },{{{{{{{{\bf{V}}}}}}}}}^{* })=&\arg \mathop{\min }\limits_{{{{{{{{{\bf{U}}}}}}}}}_{1},{{{{{{{{\bf{V}}}}}}}}}_{1},{{{{{{{\bf{V}}}}}}}}}\mathop{\sum }\limits_{j=1}^{P}\parallel {{{{{{{{\bf{y}}}}}}}}}_{j}-{{{{{{{{\bf{V}}}}}}}}}_{1}{{{{{{{{\bf{U}}}}}}}}}_{1}^{T}{{{{{{{{\bf{y}}}}}}}}}_{j}-{{{{{{{\bf{V}}}}}}}}{({{{{{{{{\bf{U}}}}}}}}}_{1}^{T}{{{{{{{{\bf{y}}}}}}}}}_{j})}^{2:M}{\parallel }^{2},\\ &{{{{{{{{\bf{U}}}}}}}}}_{1}^{T}{{{{{{{{\bf{U}}}}}}}}}_{1}={{{{{{{\bf{I}}}}}}}}.\end{array}$$

The simplest solution to this problem is **U**_1_ = **V**_1_ with the additional constraint ${{{{{{{{\bf{V}}}}}}}}}_{1}^{T}{{{{{{{\bf{V}}}}}}}}={{{{{{{\bf{0}}}}}}}}$, which represents a basic nonlinear extension of the principal component analysis^67^.

The above graph-style parametrization of the SSM breaks down for larger **y** values if ${{{{{{{{\mathcal{M}}}}}}}}}_{0}$ develops a fold over ${T}_{0}{{{{{{{{\mathcal{M}}}}}}}}}_{0}$. That creates an issue for model reduction if a nontrivial steady state on ${{{{{{{{\mathcal{M}}}}}}}}}_{0}$ falls outside the fold, as the limit cycle does in our vortex shedding example. In that case, alternative parametrization methods for ${{{{{{{{\mathcal{M}}}}}}}}}_{0}$ can be used to enhance the domain of the SSM-reduced model. These methods include selecting the columns of **U**_1_ to be the leading POD modes of the nontrivial steady state, or enlarging the embedding space with (further) delayed observations. In these cases, the columns of **V**_1_ are still orthonormal vectors spanning ${T}_{{{{{{{{\bf{0}}}}}}}}}{{{{{{{{\mathcal{M}}}}}}}}}_{0}.$

In both panels (c) of Figs. 4, 6, the SSM, ${{{{{{{{\mathcal{M}}}}}}}}}_{0}$, is nearly flat in the delay-embedding space. This turns out to be a universal property of delay embedding for small delays and low embedding dimensions (see the Supplementary Information).

For *ϵ* > 0 small (i.e., for moderate forcing), the autonomous SSM, ${{{{{{{{\mathcal{M}}}}}}}}}_{0}$, already captures the bulk nonlinear behavior of system (1). Indeed, for this forcing range, the reduced dynamics on the corresponding SSM can simply be computed as an additive perturbation of the autonomous dynamics on ${{{{{{{{\mathcal{M}}}}}}}}}_{0}$^47,68,69^ (see section “Predicting forced dynamics from unforced data”).

### SSM dynamics via extended normal forms

For an autonomous SSM ${{{{{{{{\mathcal{M}}}}}}}}}_{0}$, the reduced dynamics is governed by a vector field

19 $$\dot{{{{{{{{\boldsymbol{\eta }}}}}}}}}={{{{{{{\bf{r}}}}}}}}({{{{{{{\boldsymbol{\eta }}}}}}}})$$

with a flow map ${{{{{{{{\boldsymbol{\varphi }}}}}}}}}_{{{{{{{{\bf{r}}}}}}}}}^{t}({{{{{{{\boldsymbol{\eta }}}}}}}})$. We can generically assume that the Jacobian *D***r**(***0***) is semisimple, i.e., *D***r**(***0***)**B** = **BΛ**, where ${{{{{{{\boldsymbol{\Lambda }}}}}}}}\in {{\mathbb{C}}}^{d\times d}$ is a diagonal matrix containing the eigenvalues of *D***r**(***0***). Classic normal form theory would seek to simplify the reduced dynamics (19) in a vicinity of ***η*** = **0** via a nonlinear change of coordinates, ***η*** = **h**(**z**), so that the transformed vector field $\dot{{{{{{{{\bf{z}}}}}}}}}={{{{{{{\bf{n}}}}}}}}({{{{{{{\bf{z}}}}}}}})$ with flow map ${{{{{{{{\boldsymbol{\varphi }}}}}}}}}_{{{{{{{{\bf{n}}}}}}}}}^{t}({{{{{{{\bf{z}}}}}}}})$ has a diagonal linear part and has as few nonlinear terms in its Taylor expansion as possible. In our present setting, the origin is assumed hyperbolic, in which case the classic normal form is simply $\dot{{{{\bf{z}}}}}={{\mathbf{\Lambda}}}{{{\bf{z}}}}$ under appropriate non-resonance conditions that generically hold^40^. The corresponding normal form transformation **h**(**z**), however, is only valid on a small enough domain in which the dynamics is linearizable.

To capture non-linearizable behavior, we employ extended normal forms motivated by those used to unfold bifurcations^33^. In this approach, we construct normal forms that do not remove those polynomial terms from (19) whose removal would result in small denominators in the Taylor coefficients **h**(**z**) and hence decrease its domain of convergence. Instead, we seek a normal form for (19) of the form

20 $$\begin{array}{l}{{{{{{{\bf{n}}}}}}}}({{{{{{{\bf{z}}}}}}}};{{{{{{{\bf{N}}}}}}}})={{{{{{{\boldsymbol{\Lambda }}}}}}}}{{{{{{{\bf{z}}}}}}}}+{{{{{{{\bf{N}}}}}}}}{{{{{{{{\bf{z}}}}}}}}}^{2:N},\\ {{{{{{{\bf{h}}}}}}}}({{{{{{{\bf{z}}}}}}}};{{{{{{{\bf{H}}}}}}}})={{{{{{{\bf{B}}}}}}}}({{{{{{{\bf{z}}}}}}}}+{{{{{{{\bf{H}}}}}}}}{{{{{{{{\bf{z}}}}}}}}}^{2:N}),\,\,\,\,\,\,{{{{{{{{\bf{h}}}}}}}}}^{-1}({{{{{{{\boldsymbol{\eta }}}}}}}};{{{{{{{{\bf{H}}}}}}}}}_{{{{{{{{\boldsymbol{\star }}}}}}}}})={{{{{{{{\bf{B}}}}}}}}}^{-1}{{{{{{{\boldsymbol{\eta }}}}}}}}+{{{{{{{{\bf{H}}}}}}}}}_{\star }{({{{{{{{{\bf{B}}}}}}}}}^{-1}{{{{{{{\boldsymbol{\eta }}}}}}}})}^{2:N},\end{array}$$

where the matrices **N**, **H** and **H**_⋆_ contain the coefficients for the appropriate *d*-variate monomials. To identify near-resonances, we let **S**^2:*N*^ be the matrix of integers whose columns are the powers of the *d*-variate monomials from order 2 to *N*. We then define a matrix **Δ**^2:*N*^ containing all relevant integer linear combinations of eigenvalues as follows:

21 $${({{{{{{{{\boldsymbol{\Delta }}}}}}}}}^{2:N})}_{j,k}={({{{{{{{\rm{Im}}}}}}}}{{{{{{{\boldsymbol{\Lambda }}}}}}}})}_{j,j}-\mathop{\sum }\limits_{s=1}^{d}{({{{{{{{\rm{Im}}}}}}}}{{{{{{{\boldsymbol{\Lambda }}}}}}}})}_{s,s}{({{{{{{{{\bf{S}}}}}}}}}^{2:N})}_{s,k}.$$

Following the approach used in universal unfolding principles^41^, we collect in a set *S* the row and column indices of the entries of **Δ**^2:*N*^ for which near-resonances occur, i.e., for which the corresponding entry of **Δ**^2:*N*^ is smaller in norm than a small, preselected threshold. (The default threshold is 10^−8^ in SSMLearn.) The entries of **H** and **H**_⋆_ with indices contained in *S* are then set to zero but the corresponding monomial terms are retained in **n**(**z**; **N**). Conversely, coefficients of non-near-resonant entries of **H** and **H**_⋆_ are selected in a way so that the corresponding non–near-resonant monomials vanish from the normal form **n**(**z**; **N**). As a result, the matrix **N** is sparse, containing only the coefficients of essential, near-resonant monomials.

For example, if *d* = 2, *N* = 3 and the eigenvalues of *D***r**(***0***) form a complex pair *λ* = *α*_0_ ± *iω*_0_ with ${\omega }_{0}={{{{{{{\mathcal{O}}}}}}}}(1)$, then we have

22 $${{{{{{{{\bf{S}}}}}}}}}^{2:N}	=\left[\begin{array}{lllllll}2&1&0&3&2&1&0\\ 0&1&2&0&1&2&3\end{array}\right],\,\,{{{{{{{{\boldsymbol{\Delta }}}}}}}}}^{2:N}\\ 	=\left[\begin{array}{lllllll}-{\omega }_{0}&{\omega }_{0}&3{\omega }_{0}&-2{\omega }_{0}&0&2{\omega }_{0}&4{\omega }_{0}\\ -3{\omega }_{0}&-{\omega }_{0}&{\omega }_{0}&-4{\omega }_{0}&-2{\omega }_{0}&0&2{\omega }_{0}\end{array}\right].$$

Only two elements of **Δ**^2:*N*^ are (near-) zero, and hence the reduced dynamics in extended normal form will require learning the following coefficients:

23 $${{{{{{{{\bf{H}}}}}}}}}_{\star }	=\left[\begin{array}{lllllll}{h}_{20}&{h}_{11}&{h}_{02}&{h}_{30}&0&{h}_{12}&{h}_{03}\\ {\bar{h}}_{02}&{\bar{h}}_{11}&{\bar{h}}_{20}&{\bar{h}}_{03}&{\bar{h}}_{12}&0&{\bar{h}}_{30}\end{array}\right],\,\,\,\,\\ {{{{{{{\bf{N}}}}}}}}	=\left[\begin{array}{lllllll}0&0&0&0&{h}_{21}&0&0\\ 0&0&0&0&0&{\bar{h}}_{21}&0\end{array}\right].$$

The corresponding cubic polar form (5) is then obtained from the relations **z** = (*ρe*^*iθ*^, *ρe*^−*iθ*^) and *h*_21_ = *β* + *iγ*.

For a data-driven construction of the extended normal form (20), we first obtain an estimate for the Jacobian *D***r**(***0***) from linear regression. This determines the matrix **B** and the types of monomials arising in **h**^−1^ and **n**. Next, we note that the flow map ${{{{{{{{\boldsymbol{\varphi }}}}}}}}}_{{{{{{{{\bf{r}}}}}}}}}^{t}$ of the SSM-reduced dynamics and the flow map ${{{{{{{{\boldsymbol{\varphi }}}}}}}}}_{{{{{{{{\bf{n}}}}}}}}}^{t}$ of the extended normal form are connected through the conjugacy relationship ${{{{{{{{\boldsymbol{\varphi }}}}}}}}}_{{{{{{{{\bf{n}}}}}}}}}^{t}={{{{{{{{\bf{h}}}}}}}}}^{-1}\circ {{{{{{{{\boldsymbol{\varphi }}}}}}}}}_{{{{{{{{\bf{r}}}}}}}}}^{t}\circ {{{{{{{\bf{h}}}}}}}}$. We find the nonzero complex coefficients of **h**^−1^ and **n** by minimizing the error in this exact conjugacy over the available *P* data points, represented in the ***η*** coordinates. Specifically, we determine the nonzero elements of **H**_⋆_ and **N** as

24 $$({{{{{\bf{H}}}}}}_{\star }^{* },{{{{{{\bf{N}}}}}}}^{* })= \arg {\mathop{\min }\limits_{{{{{{\mathbf{H}}}}}}_{\star }}},{{{{{\mathbf{N}}}}}}{\mathop{\sum }\limits_{j=1}^{P}}\Vert \frac{d}{dt}{{{{{{\bf{h}}}}}}}^{-1}({{{{{{\boldsymbol{\eta }}}}}}}_{j};{{{{{{\mathbf{H}}}}}}_{\star }})-{{{{{{{\bf{n}}}}}}}}({{{{{{{{\bf{h}}}}}}}}}^{-1}({{{{{{{{\boldsymbol{\eta }}}}}}}}}_{j};{{{{{{\bf{H}}}}}}_{\star }});{{{{{{{\bf{N}}}}}}}}){\Vert}^{2},\\ {({{{{{{{\bf{N}}}}}}}})}_{s,k}=0,\,\,\forall (s,k)\in S;{({{{{{{\mathbf{H}}}}}}_{\star }})}_{s,k}=0,\forall (s,k)\,\notin\, S.$$

Once **h**^−1^ is known, we obtain the coefficients **H** of **h** via regression.

As initial condition for the minimization problem (24), we set all unknown coefficients to zero. This initial guess assumes linear dynamics, which the minimization corrects as needed. We can compute the time derivative in (24) reliably using finite differences, provided that the sampling time Δ*t* of the trajectory data is small compared to the fastest timescale of the SSM dynamics. For larger sampling times, one should use the discrete formulation of SSM theory, as discussed in section “Existence of SSMs” and^29^. In that formulation, the conjugacy error must be formulated for the 1-step prediction error of the normal form flow map ${{{{{{{{\boldsymbol{\varphi }}}}}}}}}_{{{{{{{{\bf{n}}}}}}}}}^{{{\Delta }}t}({{{{{{{\bf{z}}}}}}}})$. The matrix defined in eq. (21) also carries over to the discrete time setting, with **Λ** defined as the diagonal matrix of the logarithms of the eigenvalues of $D{{{{{{{{\boldsymbol{\varphi }}}}}}}}}_{{{{{{{{\bf{r}}}}}}}}}^{{{\Delta }}t}({{{{{{{\boldsymbol{0}}}}}}}})$.

### Prediction of forced response from unforced training data

Forced SSMs continue to be embedded in our observable space, provided that we also include the phase of the forcing among our observables^32^. (In the simplest case of periodic forcing, this inclusion is not necessary, as we pointed out Section “Embedding SSMs via generic observables”). The quasiperiodic SSM-reduced normal form of system (1) in the observable space takes the general form

25 $$\begin{array}{c}{\dot{\rho }}_{j}={\alpha }_{j}({{{{{{{\boldsymbol{\rho }}}}}}}},{{{{{{{\boldsymbol{\theta }}}}}}}}){\rho }_{j}-\mathop{\sum}\limits_{{{{{{{{\bf{k}}}}}}}}\in {K}_{j}^{\pm }}{f}_{j,{{{{{{{\bf{k}}}}}}}}}\sin \left(\langle {{{{{{{\bf{k}}}}}}}},{{{{{{{\boldsymbol{\Omega }}}}}}}}\rangle t+{\phi }_{j,{{{{{{{\bf{k}}}}}}}}}\mp {\theta }_{j}\right),\\ \!\!\!\!\!\!{\dot{\theta }}_{j}={\omega }_{j}({{{{{{{\boldsymbol{\rho }}}}}}}},{{{{{{{\boldsymbol{\theta }}}}}}}})+\mathop{\sum}\limits_{{{{{{{{\bf{k}}}}}}}}\in {K}_{j}^{\pm }}\frac{{f}_{j,{{{{{{{\bf{k}}}}}}}}}}{{\rho }_{j}}\cos \left(\langle {{{{{{{\bf{k}}}}}}}},{{{{{{{\boldsymbol{\Omega }}}}}}}}\rangle t+{\phi }_{j,{{{{{{{\bf{k}}}}}}}}}\mp {\theta }_{j}\right),\end{array}\,\,\,\,\,\,\,j=1,2,...m,\,\,\,\,\,\,{{{{{{{\bf{k}}}}}}}}\in {{\mathbb{Z}}}^{\ell },\,\,\,\,\,\,{{{{{{{\boldsymbol{\Omega }}}}}}}}\in {{\mathbb{R}}}_{+}^{\ell },$$

where the terms *f*_*j*,**k**_ and *ϕ*_*j*,**k**_ are the forcing amplitudes and phases for each mode of the SSM and for each forcing harmonic 〈**k**, **Ω**〉, while ${K}_{j}^{\pm }$ are the set containing the indexes **k** of the resonant forcing frequencies for mode *j* (see the Supplementary Information). The normal form (25) will capture non-linearizable dynamics arising from resonant interactions between the eigenfrequencies of the spectral subspace *E* (which may also contain internal resonances) and the external forcing frequencies in **Ω**. One can use numerical continuation^70^ to find nontrivial co-existing steady states (such as periodic orbits and invariant tori) in eq. (25) under varying forcing amplitudes and forcing frequencies.

To predict forced response from the SSM-based model trained on unforced data, the forcing amplitude *f* relevant for eq. (7) in the observable space needs to be related to the forcing amplitude $\left|\epsilon {{{{{{{{\bf{f}}}}}}}}}_{1}\right|$ relevant for system (1) in the physical phase space. This involves (1) employing a single forcing amplitude-frequency pair $\left(\left|\epsilon {{{{{{{{\bf{f}}}}}}}}}_{1}\right|,{{\Omega }}\right)$ in the experiment (2) measuring the periodic observable response **y**(*t*) (3) computing the corresponding normalized reduced and normalized response amplitude *ρ*_0_ (4) substituting *ρ*_0_ into the first formula in (8) and (5) solving for *f* in closed form. This *f* can then be used to make a prediction for the full FRC and response phase via (8) in the experiment for arbitrary Ω forcing frequencies. The predicted FRC may have several connected components, including isolated responses (isolas) that are notoriously difficult to detect by numerical or experimental continuation^68^.

### Summary of the algorithm

The data-driven model reduction method used in this paper is available in the open-source MATLAB^®^ package SSMLearn. User input is the measured trajectory data of the autonomous dynamical system (*ϵ* = 0), the SSM dimension *d*, the polynomial orders or approximation (*M*, *N*) for the SSM and for the extended normal form, as well as the type of the dynamical system (discrete or continuous). If the number of observables is not sufficient for manifold embedding, the data is automatically augmented with delays to reach the minimum embedding dimension *p* = 2*d* + 1. If the manifold learning returns poor results (due to, e.g., insufficient closeness of the data to the SSM), then the starting value of *p* can be increased until a good embedding is found. Then, the algorithm learns the SSM geometry in observable space and, after unsupervised detection of the required normal form, identifies the extended normal form of the reduced dynamics. The level of accuracy can be increased with larger polynomial orders, keeping in mind that excessive orders may lead to overfitting.

SSMLearn also offers all the tools we have used in this paper to analyze the reduced dynamics and make predictions for forced response from unforced training data. In particular, it contains the MATLAB^®^-based numerical continuation core COCO^70^. which can compute steady state and help with the design of nonlinear control strategies. In principle, there are no restrictions on the dimensions of the reduced-order model, yet the larger the SSM is, the more computationally expensive the problem becomes.

Qualitative or partial a priori knowledge of the linearized dynamics (e.g., some linearized modes and frequencies) helps in finding good initial conditions for trajectories to be used in SSMLearn. For example, the resonance decay method^56^ (which we exploited in our sloshing example), targets a specific 2-dimensional, stable SSM in laboratory experiments. This method consists of empirically isolating a resonant periodic motion on the SSM based on its locally maximal amplitude response under a forcing frequency sweep. Discontinuing the forcing will then generate transient decay towards the equilibrium in a close proximity of the SSM. For noisy data, filtering or dimensionality reduction can efficiently de-noise the data^67^, provided that the polynomial orders used for the description of the SSM and its reduced dynamics are not excessively large (see the Supplementary Information). For higher-dimensional SSMs, it is desirable to collect diverse trajectories to avoid bias towards specific motions. Good practice requires splitting the data sets into training, testing and validation parts.

#### Algorithm 1


SSMLearn


**Input parameters**: SSM dimension *d*, polynomial approximation orders (*M*, *N*), selection among discrete or continuous-time dynamics

**Input data**: measured unforced trajectories

**Output**: SSM geometry, extended normal form of reduced dynamics, predictions for forced response.

**1** Embed data in a suitable *p*-dimensional observable space with *p* > 2*d*.

**2** Identify the manifold parametrization in reduced coordinates.

**3** Estimate the normalized reduced dynamics after an automated identification of the required type of extended normal form.

**4** Run analytics and prediction of forced response on the SSM-reduced and normalized model.

## Supplementary information

Supplementary Information

## Data Availability

All data discussed in the results presented here is publicly available in the SSMLearn repository at github.com/haller-group/SSMLearn.

## Code availability

The code supporting the results presented here is publicly available in the SSMLearn repository at github.com/haller-group/SSMLearn.

## References

[1] Holmes, P.J., Lumley, J.L., Berkooz, G., & Rowley, C.W. *Turbulence, Coherent Structures, Dynamical Systems and Symmetry* 2nd edn, (Cambridge Monographs on Mechanics. Cambridge University Press, 2012).

[2] Awrejcewicz, J., Krys’ko, V.A., & Vakakis, A.F. *Order Reduction by Proper Orthogonal Decomposition (POD) Analysis*, 279–320 (Springer, Berlin, Heidelberg, 2004).

[3] Lu K (2019). Review for order reduction based on proper orthogonal decomposition and outlooks of applications in mechanical systems. Mech. Sys. Signal Proc..

[4] Brunton SL, Proctor JL, Kutz JN (2016). Discovering governing equations from data by sparse identification of nonlinear dynamical systems. Proc. Natl Acad. Sci..

[5] Mohamed, K.S. *Machine Learning for Model Order Reduction* (Springer, Cham, 2018).

[6] Daniel T, Casenave F, Akkari N, Ryckelynck D (2020). Model order reduction assisted by deep neural networks (rom-net). Adv. Model. Simul. Eng. Sci..

[7] Calka M (2021). Machine-learning based model order reduction of a biomechanical model of the human tongue. Computer Methods Prog. Biomedicine.

[8] Loiseau, J.-C., Brunton, S.L., & Noack, B.R.*From the POD-Galerkin method to sparse manifold models*, 279–320 (De Gruyter, Berlin, 2020).

[9] Karniadakis GE (2021). Physics-informed machine learning. Nat. Rev. Phys..

[10] Li S, Yang Y (2021). Data-driven identification of nonlinear normal modes via physics-integrated deep learning. Nonlinear Dyn..

[11] Fernex D, Noack BR, Semaan R (2021). Cluster-based network modeling–From snapshots to complex dynamical systems. Sci. Adv..

[12] Schmid PJ (2010). Dynamic mode decomposition of numerical and experimental data. J. Fluid Mech..

[13] Kutz, J.N., Brunton, S.L., Brunton, B.W., & Proctor, J.L. *Dynamic Mode Decomposition* (SIAM, Philadelphia, PA, 2016).

[14] Rowley CW, Mezić I, Bagheri S, Schlachter P, Henningson DS (2009). Spectral analysis of nonlinear flows. J. Fluid Mech..

[15] Mezić I (2013). Analysis of fluid flows via spectral properties of the Koopman operator. Ann. Rev. Fluid Mech..

[16] Mauroy, A., Mezić, I., & Susuki, Y. *The Koopman Operator in Systems and Control Concepts, Methodologies, and Applications: Concepts, Methodologies, and Applications* (Springer, New York, 2020).

[17] Lusch B, Kutz JN, Brunton SL (2018). Deep learning for universal linear embeddings of nonlinear dynamics. Nat. Commun..

[18] Otto SE, Rowley CW (2019). Linearly recurrent autoencoder networks for learning dynamics. SIAM J. Appl. Dynamical Syst..

[19] Kaiser E, Kutz JN, Brunton SL (2021). Data-driven discovery of koopman eigenfunctions for control. Mach. Learn.: Sci. Technol..

[20] Page J, Kerswell RR (2019). Koopman mode expansions between simple invariant solutions. J. Fluid Mech..

[21] Brunton SL, Brunton BW, Proctor JL, Kutz JN (2016). Koopman invariant subspaces and finite linear representations of nonlinear dynamical systems for control. PLoS ONE.

[22] Bagheri S (2013). Koopman-mode decomposition of the cylinder wake. J. Fluid Mech..

[23] Page J, Kerswell RR (2018). Koopman analysis of Burgers equation. Phys. Rev. Fluids.

[24] Dowell EH (1970). Panel flutter - a review of the aeroelastic stability of plates and shells. AIAA J..

[25] Abramian A, Virot E, Lozano E, Rubinstein SM, Schneider TM (2020). Nondestructive prediction of the buckling load of imperfect shells. Phys. Rev. Lett..

[26] Podder P, Mallick D, Amann A, Roy S (2016). Influence of combined fundamental potentials in a nonlinear vibration energy harvester. Sci. Rep..

[27] Orosz G, Stépán G (2006). Subcritical hopf bifurcations in a car-following model with reaction-time delay. Proc. R. Soc. A.

[28] Ashwin P, Wieczorek S, Vitolo R, Cox P (2012). Tipping points in open systems: bifurcation, noise-induced and rate-dependent examples in the climate system. Philos. Trans. R. Soc. A.

[29] Haller G, Ponsioen S (2016). Nonlinear normal modes and spectral submanifolds: existence, uniqueness and use in model reduction. Nonlinear Dyn..

[30] Whitney H (1944). The self-intersections of a smooth n-manifold in 2n-space. Ann. Math..

[31] Stark J, Broomhead DS, Davies ME, Huke J (1997). Takens embedding theorems for forced and stochastic systems. Nonlinear Anal.: Theory, Methods Appl..

[32] Stark J (1999). Delay embeddings for forced systems. I. deterministic forcing. J. Nonlinear Sci..

[33] Guckenheimer, J. & Holmes, P. *Nonlinear Oscillations, Dynamical Systems and Bifurcation of Vector Fields* (Springer, New York, 1983).

[34] Fenichel N (1971). Persistence and smoothness of invariant manifolds for flows. Indiana Univ. Math. J..

[35] Kuramoto, Y. *Chemical Oscillations, Waves and Turbulence* (Springer, Berlin, 1984).

[36] Jain, S. & Haller, G. How to compute invariant manifolds and their reduced dynamics in high-dimensional finite-element models? (*Nonlinear Dyn*., 2021).

[37] Sauer T, Yorke JA, Casdagli M (1997). Embedology. J. Stat. Phys..

[38] Takens, F. Detecting strange attractors in turbulence. In D. Rand and L. Young, editors, *Dynamical Systems and Turbulence, Warwick 1980*, 366–381 (Springer Berlin Heidelberg, 1981).

[39] Poincaré, H. *Les Méthodes Nouvelles de la Mécanique Céleste*. (Gauthier-Villars et Fils, Paris, 1892).

[40] Sternberg S (1958). On the structure of local homeomorphisms of euclidean n-space, II. Am. J. Math..

[41] Murdock, J. *Normal Forms and Unfoldings for Local Dynamical Systems*. (Springer Monographs in Mathematics. Springer-Verlag New York, 2003).

[42] Ponsioen S, Pedergnana T, Haller G (2018). Automated computation of autonomous spectral submanifolds for nonlinear modal analysis. J. Sound Vib..

[43] Landau LD (1944). On the problem of turbulence. Dokl. Akad. Nauk SSSR.

[44] Stuart JT (1960). On the non-linear mechanics of wave disturbances in stable and unstable parallel flows. Part 1. The basic behaviour in plane Poiseuille flow. J. Fluid Mech..

[45] Fujimura K (1997). Centre manifold reduction and the Stuart-Landau equation for fluid motions. Proc.: Math., Phys. Eng. Sci..

[46] Szalai R, Ehrhardt D, Haller G (2017). Nonlinear model identification and spectral submanifolds for multi-degree-of-freedom mechanical vibrations. Proc. R. Soc. A.

[47] Breunung T, Haller G (2018). Explicit backbone curves from spectral submanifolds of forced-damped nonlinear mechanical systems. Proc. R. Soc. A.

[48] Cenedese, M., Axås, J., Yang, H., Eriten, M., & Haller, G. Data-driven nonlinear model reduction to spectral submanifolds in mechanical systems. *arXiv:2110.01929*, 2021. 10.1098/rsta.2021.0194 PMC9207537 35719078

[49] Jain S, Tiso P, Haller G (2018). Exact nonlinear model reduction for a von Kármán beam: slow-fast decomposition and spectral submanifolds. J. Sound Vib..

[50] Barkley D, Henderson RD (1996). Three-dimensional floquet stability analysis of the wake of a circular cylinder. J. Fluid Mech..

[51] Noack BR, Afanasiev K, Morzyński M, Tadmor G, Thiele F (2003). A hierarchy of low-dimensional models for the transient and post-transient cylinder wake. J. Fluid Mech..

[52] Rowley CW, Dawson STM (2017). Model reduction for flow analysis and control. Annu. Rev. Fluid Mech..

[53] Taylor GI (1953). An experimental study of standing waves. Proc. R. Soc. Lond. Ser. A. Math. Phys. Sci..

[54] Ockendon JR, Ockendon H (1973). Resonant surface waves. J. Fluid Mech..

[55] Bäuerlein, B & Avila, K. Phase lag predicts nonlinear response maxima in liquid-sloshing experiments. *J. Fluid Mech*. **925**, 2021 (2021).

[56] Peeters M, Kerschen G, Golinval JC (2011). Dynamic testing of nonlinear vibrating structures using nonlinear normal modes. J. Sound Vib..

[57] Deng N, Noack BR, Morzyński M, Pastur LR (2020). Low-order model for successive bifurcations of the fluidic pinball. J. Fluid Mech..

[58] Shaw SW, Pierre C (1993). Normal modes for non-linear vibratory systems. J. Sound Vib..

[59] Renson L, Kerschen G, Cochelin B (2016). Numerical computation of nonlinear normal modes in mechanical engineering. J. Sound Vib..

[60] Neild SA, Champneys AR, Wagg DJ, Hill TL, Cammarano A (2015). The use of normal forms for analysing nonlinear mechanical vibrations. Philos. Trans. R. Soc. A.

[61] Cirillo GI, Mauroy A, Renson L, Kerschen G, Sepulchre R (2016). A spectral characterization of nonlinear normal modes. J. Sound Vib..

[62] Cabré X, Fontich E, de la Llave R (2003). The parameterization method for invariant manifolds i: Manifolds associated to non-resonant subspaces. Indiana Univ. Math. J..

[63] Kogelbauer F, Haller G (2018). Rigorous model reduction for a damped-forced nonlinear beam model: An infinite-dimensional analysis. J. Nonlinear Sci..

[64] Haro A, de la Llave R (2006). A parameterization method for the computation of invariant tori and their whiskers in quasi-periodic maps: rigorous results. J. Differential Eqs..

[65] Haro, A., Canadell, M., Figueras, J.-L., Luque, A., & Mondelo, J.M. *The Parameterization Method for Invariant Manifolds: from Rigorous Results to Effective Computations*. (Springer, New York, 2016).

[66] Szalai R (2020). Invariant spectral foliations with applications to model order reduction and synthesis. Nonlinear Dyn..

[67] Bishop, C.M. *Pattern Recognition and Machine Learning*. (Information Science and Statistics. Springer-Verlag New York, 2006).

[68] Ponsioen S, Pedergnana T, Haller G (2019). Analytic prediction of isolated forced response curves from spectral submanifolds. Nonlinear Dyn..

[69] Ponsioen S, Jain S, Haller G (2020). Model reduction to spectral submanifolds and forced-response calculation in high-dimensional mechanical systems. J. Sound Vib..

[70] Dankowicz, H. & Schilder, F. *Recipes for Continuation*. (Society for Industrial and Applied Mathematics, 2013).

