# Supplementary material for: Data-driven modeling and prediction of non-linearizable dynamics via spectral submanifolds
Source: Nat Commun. 2022 Feb 15;13:872. doi: 10.1038/s41467-022-28518-y (PMC8847615; doi:10.1038/s41467-022-28518-y)
Supplement: Supplementary file 1 — Supplementary Information [file 41467_2022_28518_MOESM1_ESM.pdf]

Supplementary Information for
Data-Driven Modeling and Prediction of Non-Linearizable Dynamics via Spectral Submanifolds

Mattia Cenedese[1], Joar Axås[1], Bastian Bäuerlein[2,3], Kerstin Avila[2,3] and George Haller[1,*]

[1]Institute for Mechanical Systems, ETH Zürich, Leonhardstrasse 21, 8092 Zürich, Switzerland

[2]University of Bremen, Faculty of Production Engineering, Badgasteiner Strasse 1, 28359, Bremen, Germany

[3]Leibniz Institute for Materials Engineering IWT, Badgasteiner Strasse 3, 28359, Bremen, Germany

1 Details and additional analysis for the examples

1.1 Finite-element model of a damped-forced beam

1.1.1 Setup

In our beam example [1], no body forces are present and the straight equilibrium configuration is asymptotically stable. The material properties of the beam are: the Young modulus is 70 [GPa], the density is 2700 [kg/m^3], and the Poisson ratio is 0.3. The finite-element discretization is performed using elements with cubic shape functions for the transverse deflection and linear shape functions for the axial displacement [2]. We use 12 elements, which results in convergence in static and dynamic simulations in the range of interest over uniform grid refinements. The finite element model has 33 degrees of freedom in total, including transverse displacement, axial displacements and rotations. The slowest eigenvalues of the linearized dynamics is $\lambda_1 = -3.09 \pm i657$, giving rise to a two-dimensional slow spectral subspace E_1 with spectral gap–the ratio between the real parts of the two slowest eigenvalues–equal to 7. Hence, the decay of faster linear modes is more than seven times faster than that of the slowest mode.

1.1.2 Comparison with `SSMTool`

We select the normal form order for the SSM-reduced model by minimizing the conjugacy error defined in the *Methods* section of [1]. This error is shown in Fig. 1(a) as a function of the polynomial order of approximation for the SSM, as computed by `SSMLearn`.

The training and testing trajectory data set comprises two trajectories, with the training one featuring higher amplitudes than the testing one. The conjugacy error reaches its global minimum for both trajectories at the normal-form order 9, as seen in Fig. 1(a). We nevertheless select the order 7, which produces slightly higher errors but yields a simpler reduced model.

The slow convergence in Figure 1(b) shows that the results from the `SSMTool 2.0` of [3] are approaching the boundary of the domain of convergence of the Taylor expansion for the SSM. Indeed, even for an $\mathcal{O}(15)$ approximation, the analytically predicted frequency responses do not yet match the

*Corresponding author email: georgehaller@ethz.ch

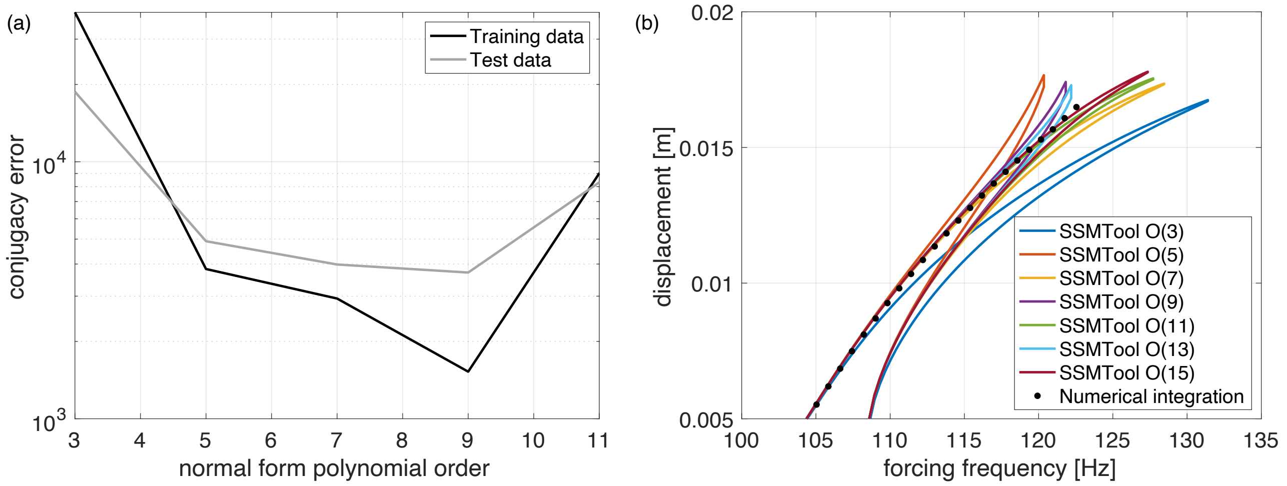

Supplementary Figure 1: (a) Conjugacy error in the data-driven construction of the extended normal form on the SSM of the damped-forced von Kármán beam as a function of the polynomial order of the normal form. (b) FRCs obtained from `SSMTool 2.0` of [3] for the beam at the highest forcing amplitude analyzed in this paper, plotted for various polynomial SSM-approximation orders. Also shown in black is the stable branch of the FRC obtained from direct numerical integration.

results from numerical integration, although the prediction error decreases under increasing polynomial order. In contrast, as already noted in our discussion of this example in [1], `SSMLearn` returns more accurate results already at $\mathcal{O}(7)$ thanks to its data-driven approach.

1.1.3 Performance under noisy observable data

To illustrate the robustness of SSM-reduced models under noise, we perturb the decaying training data of the damped beam with white noise of variance 0.5 [mm], as shown in Fig. 2(a). This emulates measurement noise or uncertainty for the observable.

The available theory of SSMs also holds approximately for finite times in this noisy setting, given that any realization of a noisy dynamical system can be approximated arbitrarily closely by a quasiperiodic dynamical system over a fixed finite time interval. The noisy attractor born out of the equilibrium under forcing can therefore be approximated by a quasiperiodic attractor with sufficiently many frequencies. Due to this multi-frequency time dependence of the corresponding SSM, we need to increase the dimensions of the delay-embedding space used for the noisy data. Accordingly, we choose to set the embedding dimension to be 200 (the scalar observable plus 199 delays) and continue to use the flat manifold approximation with an $\mathcal{O}(7)$ reduced dynamics, as we did in the case of perfect measurements. As seen in Fig. 2(b), this approach practically filters out noise from our reduced model coordinates. Indeed, the SSM-reduced model built from noisy data still reconstructs trajectories with an error of only $\mathrm{NMTE} = 7.6$ %, as shown in Figs. 2(b,c). Most importantly, the FRCs predicted from noisy data still align with the noise-free FRC obtained from direct numerical integration, as shown in Fig. 2(d).

1.2 Vortex-shedding behind a cylinder

1.2.1 Setup

For the vortex-shedding example in [1], we set the fluid kinematic viscosity to 0.01 [m^2/s] and the inflow horizontal velocity to 0.7 [m/s], resulting in a Reynolds number equal to 70. We simulate the flow using the Python-based computational platform FEniCS [4]. The mesh is formed by triangular elements of the Lagrange family; the resulting discretized model has a phase space $\mathbb{R}^n$ with dimension $n = 76876$. We integrate the flow in time using a modified version of Chorin's method [5] with time step 0.02 [s]. The linear stability analysis of the steady solution is performed via Krylov–Arnoldi iterations [6], while the POD modes (see [7]) of the limit cycle are computed using the snapshot method [8] applied to the velocities and pressures along the limit cycle. For the vortex shedding, the

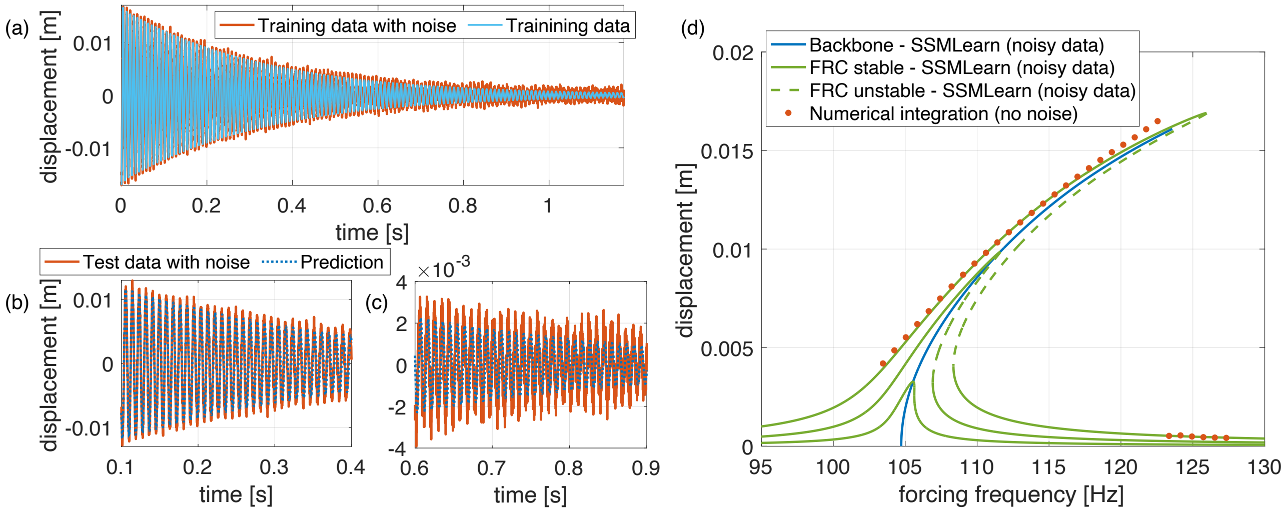

Supplementary Figure 2: (a) Noisy training trajectory and its noise-free counterpart for the von Kármán beam. (b) Noisy testing trajectory and its reconstruction from the SSM-reduced model trained on noisy data. (c) Same as (b) but zoomed in on the initial part of the time series. (d) FRCs predicted from noisy observer data and compared to direct numerical simulations in the absence of noise.

two leading POD modes capture most of the energy content of the flow on the limit cycle, as found originally by [9].

We use nine trajectories for training the SSM-reduced model, with initial conditions that are small perturbations of the steady solution along its unstable directions. Specifically, we set

$$\mathbf{x}(0) = a\mathbf{w}_{u,1}\cos\beta + a\mathbf{w}_{u,2}\sin\beta, \tag{1}$$

where $\mathbf{w}_{u,1}$, $\mathbf{w}_{u,2}$ are real unit vectors spanning the unstable subspace, $a = 0.2$ and the angle β attains 9 uniformly spaced values in the interval $[0, 2\pi)$.

1.2.2 Strong deformation along the SSM

As shown in Fig. 5(f) in [1], the SSM (unstable manifold) develops a fold over the unstable subspace of the fixed point representing the steady wake flow. Figure 3 shows additional details of this phenomenon. Trajectories initialized close to the unstable fixed point initially follow its unstable eigenspace (UE), whose real part is shown in Fig. 3(a). Continuing to evolve along the SSM, these trajectories converge to a limit cycle, whose first POD mode is shown in Fig. 3(b). As seen from a bump in the envelope of the signal in Fig. 3(c), the SSM develops a fold over UE. At the same time, there is no similar bump over the leading POD modes in Fig. 3(d), which suggest a lack of a fold over the POD modes.

The actual geometry of the fold over the UE subspace is shown in Fig. 3(e), with the SSM plotted in the space of three coordinates: the real and imaginary parts of the corresponding complex unstable eigenvector of the linearization, and the shift mode defined by [9]. In contrast, no such fold is present in Fig. 3(f), in which the same SSM is plotted using the two leading POD modes and the shift mode as coordinate axes.

1.2.3 Comparison with SINDy and DMD

Figure 4 compares results produced by `SSMLearn` with those from two leading data-driven model identification methods applied to the same dataset.

The first of these methods, SINDy [10], uses sparse regression to approximate an envisioned reduced vector field representation of a nonlinear dynamical system. As this method is not originally intended for model reduction, we examine its performance after we have projected the eight training trajectories to the two leading POD modes computed along the limit cycle of this system.

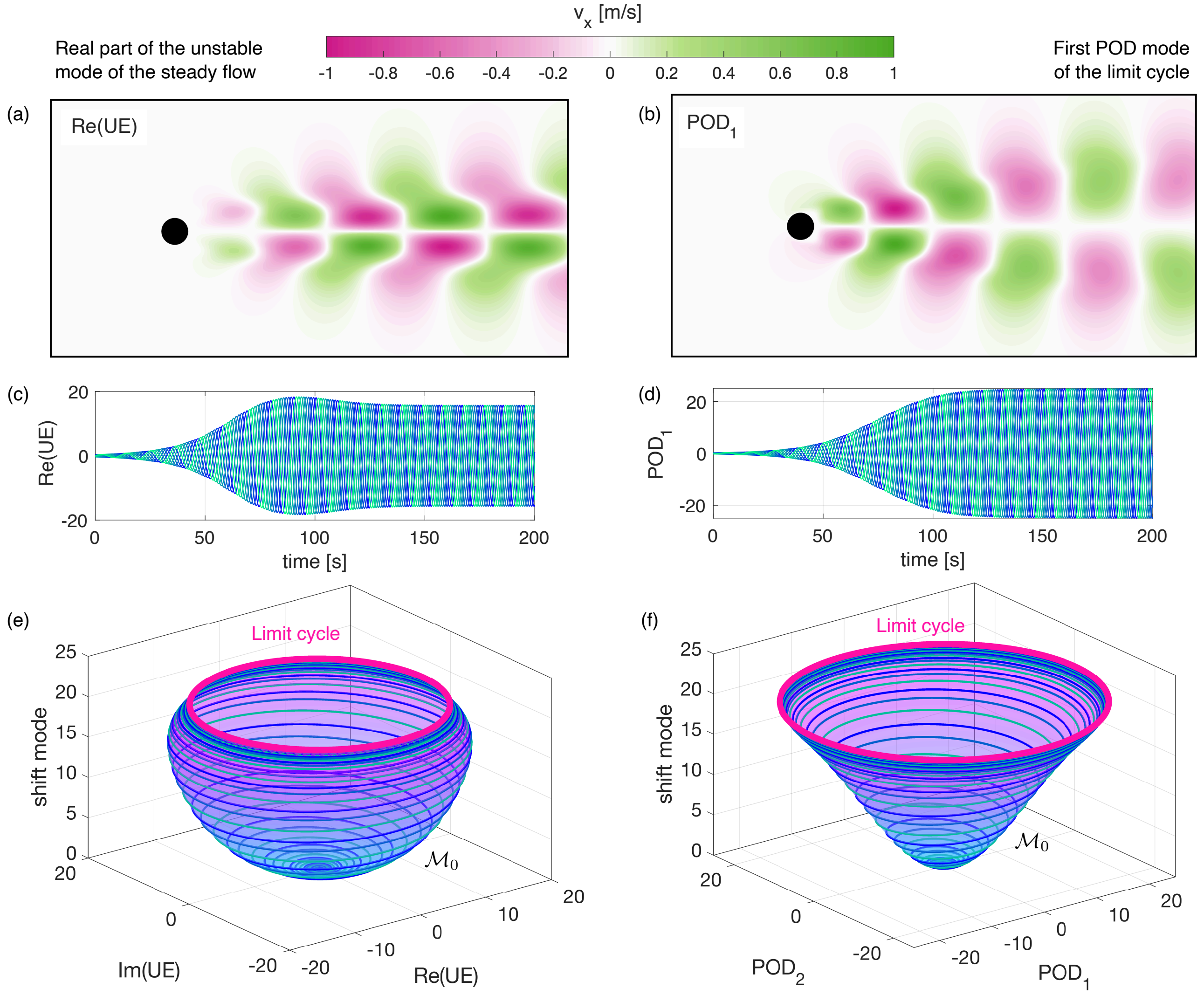

Supplementary Figure 3: Modes and coordinates for the dynamics along the SSM (unstable manifold) of the vortex shedding example. (a) Deviation of the horizontal velocity from the steady flow for the real part of the unstable mode of the unstable eigenspace. (b) Same for the first POD mode of the limit cycle. (c,d) Projection of the full trajectory onto the modes shown in (a) and (b), respectively. (e) The SSM (with some reduced trajectories) plotted over the unstable eigenspace UE and the shift mode, as defined by [9]. (f) The same SSM plotted over the two-dimensional leading POD subspace and the shift mode.

When used with polynomial regression, SINDy has two input parameters: the polynomial order and a regularizing parameter λ that controls the sparsity in the vector field to be learned. For the choice $\lambda = 3.16 \cdot 10^{-5}$, SINDy returns the sparse polynomial model

$$\begin{aligned} \dot{\eta}_1 = &\quad 0.00367 + 0.0540\eta_1 - 0.545\eta_2 - 10^{-5}\eta_1(8.77\eta_1^2 + 8.45\eta_2^2) - 10^{-4}\eta_2(1.93\eta_1^2 + 1.92\eta_2^2), \\ \dot{\eta}_2 = &\quad 0.00175 + 0.534\eta_1 + 0.0394\eta_2 - 10^{-5}\eta_2(6.32\eta_1^2 + 6.33\eta_2^2) + 10^{-4}\eta_1(2.22\eta_1^2 + 2.19\eta_2^2), \end{aligned} \quad (2)$$

where (η_1, η_2) are coordinates along the two leading POD modes of the limit cycle. Equation (2) has a sizable reconstruction error of $\mathrm{NMTE} = 108\%$, which is clearly visible in Fig. 4(a). [11] obtain comparably large errors for the same problem after applying SINDy to a latent (i.e., reduced) representation of the dynamics generated by an auto-encoder.

Cross-validation yields the best hyper-parameter value $\lambda = 1.08 \cdot 10^{-8}$, which reduces the reconstruction error to $\mathrm{NMTE} = 10.2\,\%$. The resulting model returned by SINDy, however, is dense: all its polynomial coefficients (up to degree 5) are nonzero. This is to be contrasted with the reconstruction error $\mathrm{NMTE} = 3.86\ \%$ of the sparse SSM-reduced model of [1]. The simultaneous sparsity and accuracy of the SSM-reduced model illustrates the benefits of exploiting the precise knowledge of the phase space geometry of the underlying dynamical system.

As a second method to compare with `SSMLearn`, we select the DMD [12, 13]. Because this al-

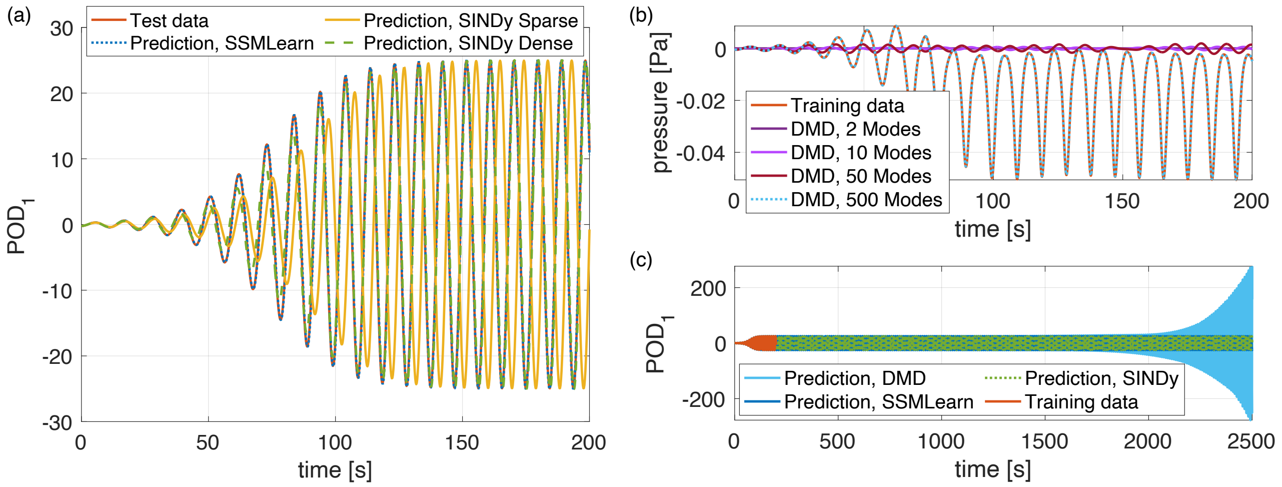

Supplementary Figure 4: Comparison of `SSMLearn` with two popular data-driven model identification methods on the vortex shedding problem. (a) Comparison of the predictions of `SSMLearn` and SINDy for the projection of the model testing trajectory to the first POD mode. (b) Reconstruction of the pressure history along the training data (see Fig. 5e of the paper) via DMD using various numbers of DMD modes. (c) Predictions from the three reduced-order models (`SSMLearn` at order 11, SINDy dense, DMD with 500 modes) for the training trajectory over a longer time interval that exceeds the training window.

gorithm fundamentally assumes near-linear dynamics, using several training trajectories with their different levels of nonlinearity leads to poor reduced models. To improve the performance of DMD, we therefore use a single training trajectory with sizable displacements. Figure 4(b) shows that while low-rank DMD fits fail to predict the trajectory from the initial condition, the DMD fit becomes very accurate for 500 modes. This fundamentally linear DMD model, however, cannot capture the limit cycle, as evidenced by the ultimate convergence of the DMD prediction to infinity. This is seen in Fig. 4(c), which shows the predictions for the testing trajectory from all three approaches over a longer time interval.

1.3 Fluid sloshing experiment

Higher-amplitude forcing not considered in [1] can lead to an apparent $1:3$ resonance in the forced response of the fluid surface [14]. `SSMLearn` is fully equipped to handle such a resonant interaction, but the available experimental data does not provide sufficient information about the higher modes involved in the interaction. More focused resonance decay experiments exciting those higher modes at higher amplitudes are needed for `SSMLearn` to construct an accurate reduced model on a four-dimensional, resonant SSM. This is the subject of our ongoing work, to be reported elsewhere.

The four-dimensional SSM-reduced model is expected to comprise a second degree of freedom that is weakly coupled to our current two-dimensional SSM model (eq. (12) in [1]). Indeed, the two-dimensional SSM model in [1] already captures the resonant branch of the FRC curve family with surprisingly high overall accuracy (see Fig. 5). This one-degree-of-freedom model, however, cannot account for the slight periodic variation in the experimentally measured response amplitudes, manifested by the three recurrent, distinct vertical dots in Fig. 5 for each forcing frequency in the resonant domain.

1.3.1 Forced response via DMD

To demonstrate that sloshing, as a non-linearizable problem, lies outside the applicability of linear data-driven modeling, we also attempt to predict the forced response using DMD [12, 13]. To help DMD discover different modal contents arising from nonlinearities, we choose a longer delay of 10 time steps with a delay-embedding dimension of 20 to create a characteristically non-flat SSM in the embedding space (see Section 2 for related information). This choice results in a close match of the decaying training signal with four DMD modes as shown in Fig. 6(a). This multimodal linear DMD

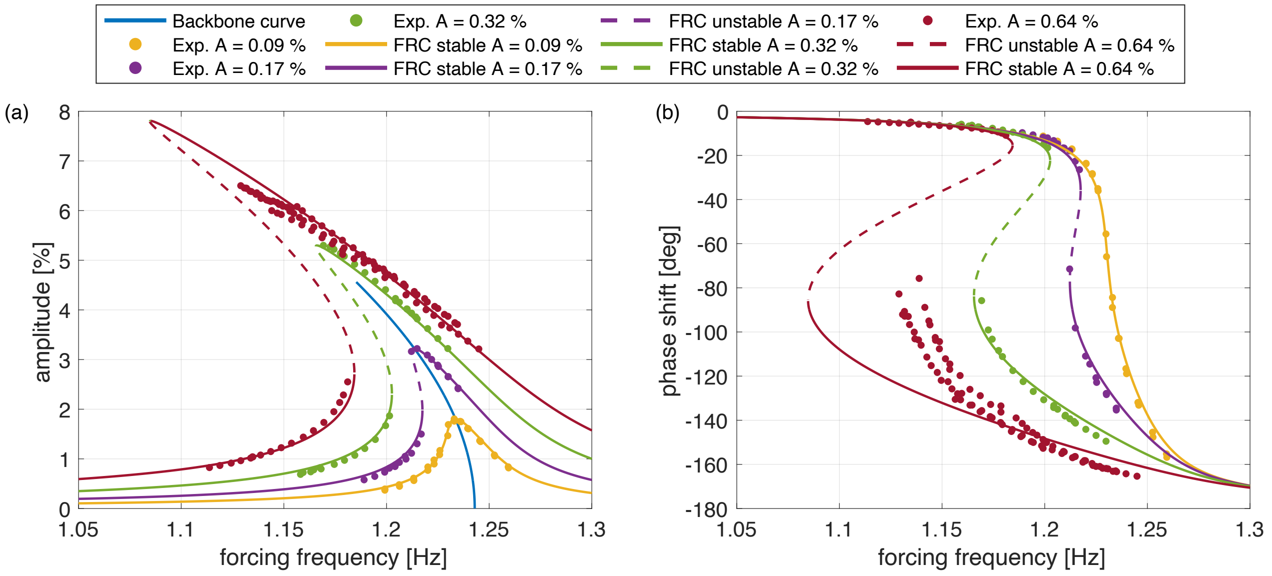

Supplementary Figure 5: Inclusion of period-three resonant response (red dots) observed from the fluid sloshing experiment for amplitude (a) and phase (b). Red lines indicate the predicted response from the two-dimensional SSM calculation at the same forcing level.

model approximates the nonlinear signal as a superposition of high- and low-amplitude trajectory patterns (see, e.g., [15]).

To obtain a forced-response prediction from this DMD model, we simply add an appropriate forcing vector to the discrete, linear, unforced DMD model and iterate the resulting inhomogeneous linear difference equation to obtain its steady-state solution. We obtain the forcing vector used for this calculation by projecting a delay-embedded harmonic load signal onto the four DMD modes. The magnitude of the modal forcing is calibrated to the known experimental forced response curves at the lowest amplitude point in the frequency sweep. The predicted periodic response amplitudes obtained from the 4-mode forced DMD model are shown in Fig. 6(b) for the same forcing amplitudes and frequencies used in Fig. 5(a). The phase response predicted by DMD can also be computed using different approximations and assumptions but yield widely differing results, which we do not show here.

For very small forcing amplitudes, the sloshing dynamics is close to linear. Accordingly, the prediction from the 4-mode forced DMD model is adequate, as illustrated by the lowest amplitude response curve in Fig. 6(b). The forced response predicted by the forced DMD model, however, completely misses the softening nonlinearity of the forced response curve even for moderate forcing amplitudes, let alone for large ones. This is no surprise, given that the overhangs in the experimental forced response curves signal the coexistence of multiple isolated stationary states, i.e., non-linearizable dynamics.

2 Geometry of SSMs in delay-embedding spaces

Both the first and the third example in [1] involves single scalar observables, and hence the embedding space for the SSMs in these examples need to be constructed from delayed measurements (see the *Methods* of [1]). As seen in Figs. 4 and 6 of [1], the SSM in the delay-embedding space is close to a plane in both cases. As we will show next, this flatness of the delay-embedding of the SSM turns out to be a general phenomenon if a moderate number of small enough delays are employed in the embedding of a signal that does not change too abruptly.

Specifically, for any smooth scalar time series, $s(t)$, and any integer k, we can write

$$s(t + k\Delta t) = s(t) + \dot{s}(t)k\Delta t + \mathcal{O}(\Delta t^2). \tag{3}$$

As a consequence, a p-dimensional delay embedding vector, $\mathbf{y} \in \mathbb{R}^p$, constructed from $s(t)$ can be

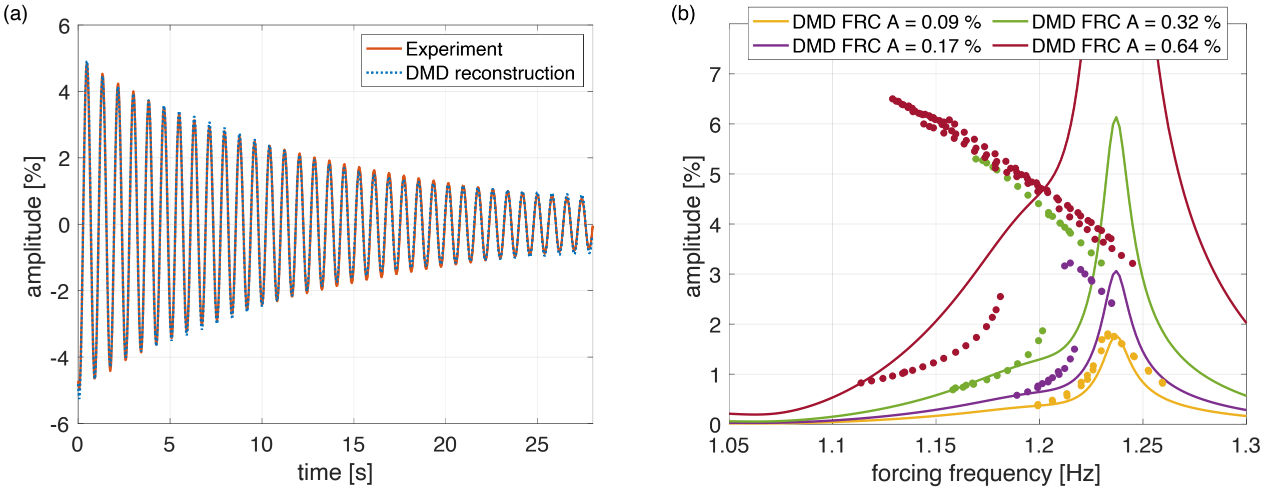

Supplementary Figure 6: Attempted prediction of the forced sloshing response from a DMD model fitted to unforced data. (a) With a high enough delay embedding dimension, DMD accurately matches the decaying center-of-mass signal of the unforced fluid mass. (b) Adding calibrated forcing to the DMD model, however, will always yield linear response. While approximately correct for very low forcing amplitudes, the forced DMD prediction therefore necessarily fails at higher amplitudes in the non-linearizable regime.

written as

$$\mathbf{y}(t) = \begin{pmatrix} s(t) \\ s(t+\Delta t) \\ s(t+2\Delta t) \\ \vdots \\ s(t+(p-1)\Delta t) \end{pmatrix} = s(t) \begin{pmatrix} 1 \\ 1 \\ 1 \\ \vdots \\ 1 \end{pmatrix} + \dot{s}(t) \begin{pmatrix} 0 \\ 1 \\ 2 \\ \vdots \\ p-1 \end{pmatrix} \Delta t + \mathcal{O}\left((p-1)^2 \Delta t^2\right). \tag{4}$$

If the time step Δt and the number of delays p is small enough in (4), then $\mathbf{y}(t)$ always lies approximately on a plane spanned by two constant vectors. In that case, therefore, a flat approximation to the SSM will always be sufficient, as long as the higher-order derivatives of $s(t)$ are not too large, i.e., the signal does not change too abruptly relative to the sampling time scale Δt. This general conclusion, however, does not imply that the reduced dynamics on the nearly flat SSMs are linear or that the SSM is nearly flat in the phase space of the underlying dynamical system.

3 The quasiperiodically forced, extended SSM normal form

We recall the main subject of [1], an n-dimensional dynamical system of the form

$$\dot{\mathbf{x}} = \mathbf{A}\mathbf{x} + \mathbf{f}_0(\mathbf{x}) + \epsilon \mathbf{f}_1(\mathbf{x}, \mathbf{\Omega}t; \epsilon), \qquad \mathbf{f}_0(x) = \mathcal{O}(|x|^2), \qquad 0 \le \epsilon \ll 1, \tag{5}$$

with a constant, semisimple matrix $\mathbf{A} \in \mathbb{R}^{n\times n}$, and with the class C^r functions $\mathbf{f}_0 \colon \mathcal{U} \to \mathbb{R}^n$ and $\mathbf{f}_1 \colon \mathcal{U} \times \mathbb{T}^\ell \to \mathbb{R}^n$, where $\mathbb{T}^\ell = S^1 \times \ldots \times S^1$ is the ℓ-dimensional torus and $\mathbf{\Omega} \in \mathbb{R}^l_+$. The assumed degree of smoothness for the right-hand side of (5) is $r \in \mathbb{N}^+ \cup \{\infty, a\}$ [16].

We define the matrix $\mathbf{T} \in \mathbb{C}^{n\times n}$ whose columns are the eigenvectors of $\mathbf{A}$. We also let $\mathbf{\Lambda} \in \mathbb{C}^{n\times n}$ denote the diagonal matrix of the corresponding eigenvalues, ordered with decreasing real parts. We write the Fourier expansion of the quasiperiodic forcing $\mathbf{f}_1(\mathbf{x}, \mathbf{\Omega}t; \epsilon)$ in the form

$$\mathbf{f}_1(\mathbf{x}, \mathbf{\Omega}t; \epsilon) = \sum_{\mathbf{k}\in\mathbb{Z}^l} \mathrm{Re}\left(\mathbf{f}^1_{\mathbf{k}} e^{i\langle \mathbf{k}, \mathbf{\Omega}\rangle t}\right) + \mathcal{O}(\epsilon\|\mathbf{x}\|) = \mathbf{T}\mathbf{g}_1(\mathbf{\Omega}t) + \mathcal{O}(\epsilon\|\mathbf{x}\|), \quad \mathbf{f}^1_{\mathbf{k}} \in \mathbb{C}^n, \tag{6}$$

with $\mathbf{g}_1(\mathbf{\Omega}t) := \mathbf{T}^{-1}\mathbf{f}_1(\mathbf{x}, \mathbf{\Omega}t; 0)$. Using the modal coordinates $\mathbf{q} \in \mathbb{C}^{n\times n}$ defined by the relation $\mathbf{x} = \mathbf{T}\mathbf{q}$, we rewrite eq. (5) as the autonomous system

$$\begin{aligned} \dot{\mathbf{q}} &= \mathbf{\Lambda}\mathbf{q} + \mathbf{g}_0(\mathbf{q}) + \epsilon\mathbf{g}_1(\varphi) + \mathcal{O}(\epsilon\|\mathbf{q}\|), \\ \dot{\varphi} &= \mathbf{\Omega}, \end{aligned} \tag{7}$$

where $\mathbf{g}_0(\mathbf{q}) = \mathbf{T}^{-1}\mathbf{f}_0(\mathbf{T}\mathbf{q})$.

Focusing on the slow SSM of dimension $2m \leq n$ related to the first m complex conjugate pairs of eigenvalues (either all stable or all unstable), we can write the extended normal form of the vector field (7) in the general polar form

$$\begin{aligned}\dot{\rho}_j &= \alpha_j(\boldsymbol{\rho},\boldsymbol{\theta})\rho_j - \sum_{\mathbf{k}\in K_j^+} f_{j,\mathbf{k}} \sin(\langle \mathbf{k},\boldsymbol{\Omega}\rangle t + \phi_{j,\mathbf{k}} - \theta_j) - \sum_{\mathbf{k}\in K_j^-} f_{j,\mathbf{k}} \sin(\langle \mathbf{k},\boldsymbol{\Omega}\rangle t + \phi_{j,\mathbf{k}} + \theta_j),\\ \dot{\theta}_j &= \omega_j(\boldsymbol{\rho},\boldsymbol{\theta}) + \sum_{\mathbf{k}\in K_j^+} \frac{f_{j,\mathbf{k}}}{\rho_j} \cos(\langle \mathbf{k},\boldsymbol{\Omega}\rangle t + \phi_{j,\mathbf{k}} - \theta_j) + \sum_{\mathbf{k}\in K_j^-} \frac{f_{j,\mathbf{k}}}{\rho_j} \cos(\langle \mathbf{k},\boldsymbol{\Omega}\rangle t + \phi_{j,\mathbf{k}} + \theta_j),\end{aligned} \tag{8}$$

for $j = 1, 2, ...m$, where K_j^+ and K_j^- are the sets of resonant forcing frequencies for mode j defined as

$$\begin{aligned}K_j^+ &:= \left\{ \mathbf{k} \in \mathbb{Z}^l, \left| \lim_{\|\boldsymbol{\rho}\|\to 0} \omega_j(\boldsymbol{\rho},\boldsymbol{\theta}) - \langle \mathbf{k},\boldsymbol{\Omega}\rangle \right| \leq \delta \right\},\\ K_j^- &:= \left\{ \mathbf{k} \in \mathbb{Z}^l, \left| \lim_{\|\boldsymbol{\rho}\|\to 0} \omega_j(\boldsymbol{\rho},\boldsymbol{\theta}) + \langle \mathbf{k},\boldsymbol{\Omega}\rangle \right| \leq \delta \right\},\end{aligned} \tag{9}$$

with δ being a small tolerance. Similar normal forms in prior work [3,17–21] focus on periodic forcing in two-dimensional SSMs.

The detailed form of the expressions $\alpha_j(\boldsymbol{\rho},\boldsymbol{\theta})$ and $\omega_j(\boldsymbol{\rho},\boldsymbol{\theta})$, and the Taylor expression of the SSM carrying the normal form (8) can be determined simultaneously via a recursive solution of the partial differential equation emerging from the invariance of the SSM. To obtain this invariance equation, we first consider a yet unknown parameterization of the SSM in the form $\mathbf{q} = \mathbf{w}(\mathbf{z},\boldsymbol{\varphi};\epsilon)$ with $\mathbf{z} \in \mathbb{C}^{2m}$. The SSM-reduced dynamics will then be of the general normal form $\dot{\mathbf{z}} = \mathbf{n}(\mathbf{z},\boldsymbol{\varphi};\epsilon)$, satisfying $\mathbf{w}(\mathbf{0},\boldsymbol{\varphi};0) = \mathbf{0}$ and $\mathbf{n}(\mathbf{0},\boldsymbol{\varphi};0) = \mathbf{0}$.

If we substitute the parametrization and the reduced dynamics expressions into eq. (7), we obtain the invariance equation [22]

$$D_{\mathbf{z}}\mathbf{w}(\mathbf{z},\boldsymbol{\varphi};\epsilon)\mathbf{n}(\mathbf{z},\boldsymbol{\varphi};\epsilon) + D_{\boldsymbol{\varphi}}\mathbf{w}(\mathbf{z},\boldsymbol{\varphi};\epsilon)\boldsymbol{\Omega} = \boldsymbol{\Lambda}\mathbf{w}(\mathbf{z},\boldsymbol{\varphi};\epsilon) + \mathbf{g}_0(\mathbf{w}(\mathbf{z},\boldsymbol{\varphi};\epsilon)) + \epsilon\mathbf{g}_1(\boldsymbol{\varphi}) + \mathcal{O}(\epsilon\|\mathbf{w}\|). \tag{10}$$

By the smooth dependence of the SSM on ϵ [21], we can write $\mathbf{w}(\mathbf{z},\boldsymbol{\varphi};\epsilon) = \mathbf{w}_0(\mathbf{z}) + \epsilon\mathbf{w}_1(\mathbf{z},\boldsymbol{\varphi}) + \mathcal{O}(\epsilon^2)$ and $\mathbf{n}(\mathbf{z},\boldsymbol{\varphi};\epsilon) = \mathbf{n}_0(\mathbf{z}) + \epsilon\mathbf{n}_1(\mathbf{z},\boldsymbol{\varphi}) + \mathcal{O}(\epsilon^2)$. Substituting these expression in eq. (10) and the Taylor expansion $\mathbf{g}_0(\mathbf{w}(\mathbf{z},\boldsymbol{\varphi};\epsilon)) = \mathbf{g}_0(\mathbf{w}_0(\mathbf{z})) + \epsilon D\mathbf{g}_0(\mathbf{w}_0(\mathbf{z}))\mathbf{w}_1(\mathbf{z},\boldsymbol{\varphi}) + \mathcal{O}(\epsilon^2)$, we then equate terms of equal order in ϵ to obtain the invariance equation to be detailed next.

3.1 Solving the autonomous invariance equation

Equating terms of order $\mathcal{O}(1)$ in eq. (10), we obtain the autonomous invariance equation

$$D\mathbf{w}_0(\mathbf{z})\mathbf{n}_0(\mathbf{z}) = \boldsymbol{\Lambda}\mathbf{w}_0(\mathbf{z}) + \mathbf{g}_0(\mathbf{w}_0(\mathbf{z})). \tag{11}$$

We seek the solution of this partial differential equation via the Taylor expansions

$$\mathbf{w}_0(\mathbf{z}) = \sum_{j=1}^{M} \mathbf{W}_j^0\mathbf{z}^j, \quad \mathbf{n}_0(\mathbf{z}) = \sum_{j=1}^{M} \mathbf{N}_j^0\mathbf{z}^j, \quad \mathbf{g}_0(\mathbf{q}) = \sum_{j=2}^{M_g} \mathbf{G}_j^0\mathbf{q}^j, \tag{12}$$

where $\mathbf{z}^j$ denotes the family of all monomials in $2m$ variables of degree j, and $\mathbf{W}_j^0, \mathbf{N}_j^0$ and $\mathbf{G}_j^0$ are matrices storing the corresponding polynomial coefficients. We also introduce the notation $\mathbf{S}_{2m}^j$ for the matrix of integers whose columns are the exponents of the $2m$-variate monomials of order j, i.e., the components of $\mathbf{z}^j$. For example, if $m = 2$ and $j = 3$, then

$$\mathbf{z}^2 = (z_1^2, z_1z_2, z_1z_3, z_1z_4, z_2^2, z_2z_3, z_2z_4, z_3^2, z_3z_4, z_4^2)^T,$$
$$\mathbf{S}_{2m}^2 = \begin{bmatrix} 2 & 1 & 1 & 1 & 0 & 0 & 0 & 0 & 0 & 0\\ 0 & 1 & 0 & 0 & 2 & 1 & 1 & 0 & 0 & 0\\ 0 & 0 & 1 & 0 & 0 & 1 & 0 & 2 & 1 & 0\\ 0 & 0 & 0 & 1 & 0 & 0 & 1 & 0 & 1 & 2 \end{bmatrix}. \tag{13}$$

After substituting the expression (12) into eq. (11), we equate the terms of equal polynomial order to obtain a set of *cohomological equations* [22] to be solved recursively in j for the unknown matrices $\mathbf{W}_j^0, \mathbf{N}_j^0$. At the $j = 1$ step in this recursion, we have the cohomological equation

$$\mathbf{W}_1^0 \mathbf{N}_1^0 = \mathbf{\Lambda} \mathbf{W}_1^0, \tag{14}$$

which is solved by

$$\mathbf{W}_1^0 = \begin{bmatrix} \mathbf{I} \\ \mathbf{0} \end{bmatrix}, \quad \mathbf{N}_1^0 = \mathbf{\Lambda}_m = \mathrm{diag}(\boldsymbol{\lambda}_m), \quad \boldsymbol{\lambda}_m = (\lambda_1, \bar{\lambda}_1, \lambda_2, \bar{\lambda}_2, ..., \lambda_m, \bar{\lambda}_m)^T. \tag{15}$$

Noting that

$$D\mathbf{w}_0(\mathbf{z}) \mathbf{N}_1^0 \mathbf{z} = \sum_{j=1}^{M} \mathbf{W}_j^0 (D\mathbf{z}^j) \mathbf{N}_1^0 \mathbf{z} = \sum_{j=1}^{M} \mathbf{W}_j^0 \mathbf{\Lambda}_m^j \mathbf{z}^j, \tag{16}$$

where $\mathbf{\Lambda}_m^j = \mathrm{diag}((\mathbf{S}_{2m}^j)^T \boldsymbol{\lambda}_m)$, we can rewrite eq. (11) as

$$\sum_{j=1}^{M} \left(\mathbf{\Lambda} \mathbf{W}_j^0 - \mathbf{W}_j^0 \mathbf{\Lambda}_m^j - \mathbf{W}_1^0 \mathbf{N}_j^0 \right) \mathbf{z}^j = \left(\sum_{j=2}^{M} \mathbf{W}_j^0 (D\mathbf{z}^j) \right) \left(\sum_{j=2}^{M} \mathbf{N}_j^0 \mathbf{z}^j \right) - \sum_{j=2}^{M_g} \mathbf{G}_j^0 \left(\sum_{j=1}^{M} \mathbf{W}_j^0 \mathbf{z}^j \right)^j. \tag{17}$$

We observe that, in the right hand side of eq. (17), the coefficients for the monomials of order k only depend on the coefficients in the matrices $\mathbf{W}_j^0, \mathbf{N}_j^0$ for $j = 1, 2, ..., k-1$. This motivates us to define

$$\sum_{j=2}^{M} \mathbf{B}_j^0 \mathbf{z}^j = \left(\sum_{j=2}^{M} \mathbf{W}_j^0 (D\mathbf{z}^j) \right) \left(\sum_{j=2}^{M} \mathbf{N}_j^0 \mathbf{z}^j \right) - \sum_{j=2}^{M_g} \mathbf{G}_j^0 \left(\sum_{j=1}^{M} \mathbf{W}_j^0 \mathbf{z}^j \right)^j, \tag{18}$$

where, for example, $\mathbf{B}_2^0 \mathbf{z}^2 = \mathbf{G}_2^0 (\mathbf{W}_1^0 \mathbf{z})^2$. Therefore, the coefficients at order k must satisfy the equation

$$\mathbf{\Lambda} \mathbf{W}_k^0 - \mathbf{W}_k^0 \mathbf{\Lambda}_m^k = \mathbf{W}_1^0 \mathbf{N}_k^0 + \mathbf{B}_k^0. \tag{19}$$

Using the notation

$$\mathbf{\Lambda} = \begin{bmatrix} \mathbf{\Lambda}_m & \mathbf{0} \\ \mathbf{0} & \mathbf{\Lambda}_{out} \end{bmatrix}, \quad \mathbf{W}_k^0 = \begin{bmatrix} \mathbf{W}_{k,in}^0 \\ \mathbf{W}_{k,out}^0 \end{bmatrix}, \quad \mathbf{B}_k^0 = \begin{bmatrix} \mathbf{B}_{k,in}^0 \\ \mathbf{B}_{k,out}^0 \end{bmatrix}, \tag{20}$$

we decouple the matrix equation (19) as

$$\begin{cases} \mathbf{\Lambda}_m \mathbf{W}_{k,in}^0 - \mathbf{W}_{k,in}^0 \mathbf{\Lambda}_m^k = \mathbf{W}_1^0 \mathbf{N}_k^0 + \mathbf{B}_{k,in}^0, \\ \mathbf{\Lambda}_{out} \mathbf{W}_{k,out}^0 - \mathbf{W}_{k,out}^0 \mathbf{\Lambda}_m^k = \mathbf{B}_{k,out}^0. \end{cases} \tag{21}$$

From the second set of equations in (21), we find $\mathbf{W}_{k,out}^0$ to be

$$\left(\mathbf{W}_{k,out}^0\right)_{r,s} = \frac{\left(\mathbf{B}_{k,out}^0\right)_{r,s}}{\left(\mathbf{\Lambda}_{out}\right)_{r,r} - \left(\mathbf{\Lambda}_m^k\right)_{s,s}}, \tag{22}$$

because the denominator of this expression is nonzero by the non-resonance assumption for the existence of autonomous SSMs (see the *Methods* section of [1]). The first set of equations in (21) is underdetermined, having more unknowns than equations. We can, therefore, choose different forms for the reduced dynamics, of which we choose the extended normal-form style parametrization. This avoids small denominators in the parametrization and hence enhances its domain of convergence.

Specifically, we follow the extended normal form principle discussed in [1] to obtain

$$\begin{cases} \left(\mathbf{W}^0_{k,in}\right)_{r,s} = 0, \quad \left(\mathbf{N}^0_k\right)_{r,s} = -\left(\mathbf{B}^0_{k,in}\right)_{r,s}, & \text{if} \quad \left(\boldsymbol{\Delta}^k\right)_{r,s} \leq \delta \\ \left(\mathbf{W}^0_{k,in}\right)_{r,s} = \dfrac{\left(\mathbf{B}^0_{k,in}\right)_{r,s}}{(\boldsymbol{\Lambda}_m)_{r,r} - \left(\boldsymbol{\Lambda}^k_m\right)_{s,s}}, \quad \left(\mathbf{N}^0_k\right)_{r,s} = 0, & \text{otherwise.} \end{cases} \tag{23}$$

Here δ is a small tolerance parameter defining near-resonances among the linearized frequencies related to the SSM, and $\left(\boldsymbol{\Delta}^k\right)_{r,s}$ is defined by

$$\left(\boldsymbol{\Delta}^k\right)_{r,s} = (\mathrm{Im}\boldsymbol{\Lambda}_m)_{r,r} - \sum_{l=1}^{2m} (\mathrm{Im}\boldsymbol{\Lambda}_m)_{l,l} \left(\mathbf{S}^k_{2m}\right)_{l,s}. \tag{24}$$

We perform similar calculations recursively for $j > 1$ to obtain the higher-order polynomial terms in the parametrization and the reduced dynamics defined in eq. (12).

3.2 Solving the non-autonomous invariance equation

The terms of order $\mathcal{O}(\epsilon)$ in the invariance equation (10) must satisfy

$$D\mathbf{w}_0(\mathbf{z})\mathbf{n}_1(\mathbf{z},\boldsymbol{\varphi}) + D_{\mathbf{z}}\mathbf{w}_1(\mathbf{z},\boldsymbol{\varphi})\mathbf{n}_0(\mathbf{z}) + D_{\boldsymbol{\varphi}}\mathbf{w}_1(\mathbf{z},\boldsymbol{\varphi})\boldsymbol{\Omega} = \boldsymbol{\Lambda}\mathbf{w}_1(\mathbf{z},\boldsymbol{\varphi}) + D\mathbf{g}_0(\mathbf{w}_0(\mathbf{z}))\mathbf{w}_1(\mathbf{z},\boldsymbol{\varphi}) + \mathbf{g}_1(\boldsymbol{\varphi}).$$

The $\mathcal{O}(1)$ terms of $\mathbf{w}_1$ and $\mathbf{n}_1$ then satisfy

$$\boldsymbol{\Lambda}\mathbf{w}_1(\mathbf{0},\boldsymbol{\varphi}) - D_{\boldsymbol{\varphi}}\mathbf{w}_1(\mathbf{0},\boldsymbol{\varphi})\boldsymbol{\Omega} = \mathbf{W}^0_1\mathbf{n}_1(\mathbf{0},\boldsymbol{\varphi}) - \mathbf{g}_1(\boldsymbol{\varphi}). \tag{25}$$

The last forcing term,

$$\mathbf{g}_1(\boldsymbol{\varphi}) = \sum_{|\mathbf{k}|=1}^{\infty} \mathbf{T}^{-1}\mathbf{f}^1_{\mathbf{k}} e^{+i\langle\mathbf{k},\boldsymbol{\varphi}\rangle} + \mathbf{T}^{-1}\bar{\mathbf{f}}^1_{\mathbf{k}} e^{-i\langle\mathbf{k},\boldsymbol{\varphi}\rangle} = \sum_{|\mathbf{k}|=1}^{\infty} \mathbf{g}^+_{\mathbf{k}} e^{i\langle\mathbf{k},\boldsymbol{\varphi}\rangle} + \mathbf{g}^-_{\mathbf{k}} e^{-i\langle\mathbf{k},\boldsymbol{\varphi}\rangle}, \tag{26}$$

satisfies

$$\left(\bar{\mathbf{g}}^+_{\mathbf{k}}\right)_{2j-1} = \left(\mathbf{g}^-_{\mathbf{k}}\right)_{2j}, \quad \left(\bar{\mathbf{g}}^-_{\mathbf{k}}\right)_{2j-1} = \left(\mathbf{g}^+_{\mathbf{k}}\right)_{2j}, \quad j = 1, 2, ..., m, \tag{27}$$

due to the complex conjugate columns of the matrix $\mathbf{T}$.

Substituting the expansions

$$\begin{aligned} \mathbf{w}_1(\mathbf{z},\boldsymbol{\varphi}) &= \sum_{|\mathbf{k}|=1}^{\infty} \mathbf{w}^+_{\mathbf{k}} e^{i\langle\mathbf{k},\boldsymbol{\varphi}\rangle} + \mathbf{w}^-_{\mathbf{k}} e^{-i\langle\mathbf{k},\boldsymbol{\varphi}\rangle} + \mathcal{O}(\|\mathbf{z}\|), \\ \mathbf{n}_1(\mathbf{z},\boldsymbol{\varphi}) &= \sum_{|\mathbf{k}|=1}^{\infty} \mathbf{n}^+_{\mathbf{k}} e^{i\langle\mathbf{k},\boldsymbol{\varphi}\rangle} + \mathbf{n}^-_{\mathbf{k}} e^{-i\langle\mathbf{k},\boldsymbol{\varphi}\rangle} + \mathcal{O}(\|\mathbf{z}\|), \end{aligned} \tag{28}$$

and (26) into eq. (25), we solve for $\mathbf{w}^+_{\mathbf{k}}$ and $\mathbf{w}^-_{\mathbf{k}}$ to obtain

$$\begin{cases} \left(\mathbf{w}^+_{\mathbf{k}}\right)_r = \dfrac{\left(\mathbf{n}^+_{\mathbf{k}}\right)_r - \left(\mathbf{g}^+_{\mathbf{k}}\right)_r}{(\boldsymbol{\Lambda}_m)_{r,r} - i\langle\mathbf{k},\boldsymbol{\Omega}\rangle}, \quad \left(\mathbf{w}^-_{\mathbf{k}}\right)_r = \dfrac{\left(\mathbf{n}^-_{\mathbf{k}}\right)_r - \left(\mathbf{g}^-_{\mathbf{k}}\right)_r}{(\boldsymbol{\Lambda}_m)_{r,r} + i\langle\mathbf{k},\boldsymbol{\Omega}\rangle}, & \text{if} \quad r \leq 2m \\ \left(\mathbf{w}^\pm_{\mathbf{k}}\right)_r = \dfrac{-\left(\mathbf{g}^\pm_{\mathbf{k}}\right)_r}{(\boldsymbol{\Lambda}_m)_{r,r} \mp i\langle\mathbf{k},\boldsymbol{\Omega}\rangle} & \text{otherwise.} \end{cases} \tag{29}$$

As previously, the first $2m$ equations in eq. (29) are underdetermined. Their simplest meaningful solution yields the normalized reduced dynamics. This solution is influenced by possible resonances with the external forcing. Those resonances appear in weakly damped systems for a certain SSM mode $j \leq m$ whenever $(\mathrm{Im}\boldsymbol{\Lambda}_m)_{2j-1,2j-1} \approx \langle\mathbf{k},\boldsymbol{\Omega}\rangle$ or $(\mathrm{Im}\boldsymbol{\Lambda}_m)_{2j,2j} \approx \langle\mathbf{k},\boldsymbol{\Omega}\rangle$, where we have $(\mathrm{Im}\boldsymbol{\Lambda}_m)_{2j-1,2j-1} = -(\mathrm{Im}\boldsymbol{\Lambda}_m)_{2j,2j}$. If there are no such resonances, the forcing vectors in the reduced

dynamics $\mathbf{n}_{\mathbf{k}}^{+}, \mathbf{n}_{\mathbf{k}}^{-}$ can be chosen zero. Hence, the linearizable forced state appears in the parametrization but not in the reduced dynamics, at least not up to the current order of expansion. In contrast, non-linearizable forced states may arise due to resonances. As an alternative, we could retain all forcing terms in the reduced dynamics by setting $\left(\mathbf{n}_{\mathbf{k}}^{-}\right)_r = \left(\mathbf{g}_{\mathbf{k}}^{-}\right)_r$ and $\left(\mathbf{w}_{\mathbf{k}}^{-}\right)_r = 0$ for $r \leq 2m$, but this would lead to unnecessary complexity in the reduced model.

Considering a specific mode j, we define the sets $K_j^{\pm} \subset \mathbb{Z}^l$ of resonant indexes as in eq. (9). Adopting again the normal-form style parametrization, we impose the relationships

$$\begin{cases} \left(\mathbf{n}_{\mathbf{k}}^{+}\right)_{2j-1} = \left(\mathbf{g}_{\mathbf{k}}^{+}\right)_{2j-1}, & \left(\mathbf{n}_{\mathbf{k}}^{-}\right)_{2j} = \left(\mathbf{g}_{\mathbf{k}}^{-}\right)_{2j}, \quad \left(\mathbf{n}_{\mathbf{k}}^{-}\right)_{2j-1} = \left(\mathbf{n}_{\mathbf{k}}^{+}\right)_{2j} = 0, \quad \text{if } \mathbf{k} \in K_j^{+}, \\ \left(\mathbf{n}_{\mathbf{k}}^{-}\right)_{2j-1} = \left(\mathbf{g}_{\mathbf{k}}^{-}\right)_{2j-1}, & \left(\mathbf{n}_{\mathbf{k}}^{+}\right)_{2j} = \left(\mathbf{g}_{\mathbf{k}}^{+}\right)_{2j}, \quad \left(\mathbf{n}_{\mathbf{k}}^{+}\right)_{2j-1} = \left(\mathbf{n}_{\mathbf{k}}^{-}\right)_{2j} = 0, \quad \text{if } \mathbf{k} \in K_j^{-}, \end{cases} \tag{30}$$

while the parametrization terms follow from (29). We note that $K_j^{+} \cap K_j^{-} = \emptyset$.

3.3 SSM-reduced dynamics in polar coordinates

For the jth mode, we find that $(\mathbf{n}_0(\mathbf{z}))_{2j}$ is the complex conjugate of $(\mathbf{n}_0(\mathbf{z}))_{2j-1}$. Thus, letting $g_{j,\mathbf{k}}^{\pm} = \left(\mathbf{g}_{\mathbf{k}}^{\pm}\right)_{2j-1}$ so that $\left(\mathbf{g}_{\mathbf{k}}^{\mp}\right)_{2j} = \bar{g}_{j,\mathbf{k}}$ as in eq. (27), we obtain the reduced dynamics on the SSM in the form

$$\begin{aligned} \dot{z}_j &= (\mathbf{n}_0(\mathbf{z}))_{2j-1} + \sum_{\mathbf{k}\in K_j^{+}} \epsilon g_{j,\mathbf{k}}^{+} e^{i\langle \mathbf{k},\boldsymbol{\varphi}\rangle} + \sum_{\mathbf{k}\in K_j^{-}} \epsilon g_{j,\mathbf{k}}^{-} e^{-i\langle \mathbf{k},\boldsymbol{\varphi}\rangle} + \mathcal{O}(\epsilon\|\mathbf{z}\|), \\ \dot{\bar{z}}_j &= (\bar{\mathbf{n}}_0(\mathbf{z}))_{2j-1} + \sum_{\mathbf{k}\in K_j^{+}} \epsilon \bar{g}_{j,\mathbf{k}}^{+} e^{-i\langle \mathbf{k},\boldsymbol{\varphi}\rangle} + \sum_{\mathbf{k}\in K_j^{-}} \epsilon \bar{g}_{j,\mathbf{k}}^{-} e^{i\langle \mathbf{k},\boldsymbol{\varphi}\rangle} + \mathcal{O}(\epsilon\|\mathbf{z}\|). \end{aligned} \tag{31}$$

Introducing the polar coordinates $z_j = \rho_j e^{i\theta_j}$ and setting $g_{j,\mathbf{k}}^{\pm} = |g_{j,\mathbf{k}}^{\pm}| e^{\pm i(\phi_{j,\mathbf{k}}+\pi/2)}$, we obtain from (31) the equations

$$\begin{aligned} \dot{\rho}_j + i\rho_j\dot{\theta}_j &= e^{-i\theta_j}\left(\mathbf{n}_0(\mathbf{z})\right)_{2j-1} + \sum_{\mathbf{k}\in K_j^{\pm}} \epsilon |g_{j,\mathbf{k}}^{\pm}| e^{\pm i(\langle \mathbf{k},\boldsymbol{\varphi}\rangle + \phi_{j,\mathbf{k}} \mp \theta_j + \pi/2)} + \mathcal{O}(\epsilon\|\mathbf{z}\|), \\ \dot{\rho}_j - i\rho_j\dot{\theta}_j &= e^{i\theta_j}\left(\bar{\mathbf{n}}_0(\mathbf{z})\right)_{2j-1} + \sum_{\mathbf{k}\in K_j^{\pm}} \epsilon |g_{j,\mathbf{k}}^{+}| e^{\mp i(\langle \mathbf{k},\boldsymbol{\varphi}\rangle + \phi_{j,\mathbf{k}} \mp \theta_j + \pi/2)} + \mathcal{O}(\epsilon\|\mathbf{z}\|), \end{aligned} \tag{32}$$

where we have grouped together the $\pm$ sets for notational ease, i.e.,

$$\begin{aligned} \sum_{\mathbf{k}\in K_j^{\pm}} \epsilon |g_{j,\mathbf{k}}^{\pm}| e^{\pm i(\langle \mathbf{k},\boldsymbol{\varphi}\rangle + \phi_{j,\mathbf{k}} \mp \theta_j + \pi/2)} = & \sum_{\mathbf{k}\in K_j^{+}} \epsilon |g_{j,\mathbf{k}}^{+}| e^{i(\langle \mathbf{k},\boldsymbol{\varphi}\rangle + \phi_{j,\mathbf{k}} - \theta_j + \pi/2)} + \\ & + \sum_{\mathbf{k}\in K_j^{-}} \epsilon |g_{j,\mathbf{k}}^{-}| e^{-i(\langle \mathbf{k},\boldsymbol{\varphi}\rangle + \phi_{j,\mathbf{k}} + \theta_j + \pi/2)}. \end{aligned}$$

By separating the time derivatives of the amplitude and phase variables in (32), we obtain the normal form

$$\begin{aligned} \dot{\rho}_j &= \mathrm{Re}\left(e^{-i\theta_j}\left(\mathbf{n}_0(\mathbf{z})\right)_{2j-1}\right) - \sum_{\mathbf{k}\in K_j^{\pm}} \epsilon |g_{j,\mathbf{k}}^{\pm}| \sin(\langle \mathbf{k},\boldsymbol{\varphi}\rangle + \phi_{j,\mathbf{k}} \mp \theta_j) + \mathcal{O}(\epsilon\|\boldsymbol{\rho}\|), \\ \rho_j\dot{\theta}_j &= \mathrm{Im}\left(e^{-i\theta_j}\left(\mathbf{n}_0(\mathbf{z})\right)_{2j-1}\right) + \sum_{\mathbf{k}\in K_j^{\pm}} \epsilon |g_{j,\mathbf{k}}^{\pm}| \cos(\langle \mathbf{k},\boldsymbol{\varphi}\rangle + \phi_{j,\mathbf{k}} \mp \theta_j) + \mathcal{O}(\epsilon\|\boldsymbol{\rho}\|). \end{aligned} \tag{33}$$

With the definitions

$$\begin{aligned} \alpha_j(\boldsymbol{\rho},\boldsymbol{\theta}) &= \mathrm{Re}\left(\frac{(\mathbf{n}_0(\mathbf{z}))_{2j-1}}{z_j}\right), \\ \omega_j(\boldsymbol{\rho},\boldsymbol{\theta}) &= \mathrm{Im}\left(\frac{(\mathbf{n}_0(\mathbf{z}))_{2j-1}}{z_j}\right), \end{aligned} \tag{34}$$

and with the rescaling $f_{j,\mathbf{k}} = \epsilon|g^{\pm}_{j,\mathbf{k}}|$, eq. (33) provides the detailed form of the polar normal form given in the methods section of [1]. We also note that

$$\lim_{\|\boldsymbol{\rho}\|\to 0} \alpha_j(\boldsymbol{\rho},\boldsymbol{\theta}) + i\omega_j(\boldsymbol{\rho},\boldsymbol{\theta}) = (\boldsymbol{\Lambda}_m)_{2j-1,2j-1}\,.$$

For a two-dimensional SSM, periodically forced ($l = 1$) with a single sinusoidal term near resonance, we have $K^+ = \{1\}$, $K^- = \emptyset$. The amplitude and phase dynamics in (34) only depends on ρ, so that we recover eq. (7) of [1] by setting $\theta = \Omega t + \phi_{1,1} + \psi$.

The model reduction we have outlined for periodic and quasiperiodic SSMs features an autonomous core plus the leading-order forcing term, providing an overall $\mathcal{O}(\epsilon\|\boldsymbol{\rho}\|)$ accuracy. More accurate approximations can also be derived including the remaining $\mathcal{O}(\epsilon)$ terms that also depend on higher powers of the amplitudes $\boldsymbol{\rho}$ or higher powers of ϵ. While the leading-order reduced dynamics, $\mathbf{n}_1(\boldsymbol{\varphi})$, can already identify coexisting isolated steady states, the parametrization correction term, $\mathbf{w}_1(\boldsymbol{\varphi})$, reveals an additional, small quasiperiodic modulation to these autonomous steady states. These two terms both depend on the forcing coefficients $g_{j,\mathbf{k}}$, which can be identified via calibration to experiments. If information on modes outside those related to the slow $2m$-dimensional SSM is not available, then the reduced-order model is still accurate up to a small quasiperiodic correction if we set $\left(\mathbf{w}^{\pm}_{\mathbf{k}}\right)_{2j-1} = \left(\mathbf{w}^{\pm}_{\mathbf{k}}\right)_{2j} = 0$ for $j > m$. However, if there are resonances involving those additional modes, it is advisable to increase the dimension of the SSM by including them. This will generally require the collection of further data with additional modal content.

Supplementary References

[1] M. Cenedese, J. Axås, B. Bäuerlein, K. Avila, and G. Haller. Data-driven modeling and prediction of non-linearizable dynamics via spectral submanifolds. *Submitted*, 2021.

[2] S. Jain, P. Tiso, and G. Haller. Exact nonlinear model reduction for a von Kármán beam: slow-fast decomposition and spectral submanifolds. *Journal of Sound and Vibration*, 423:195–211, 2018.

[3] S. Jain and G. Haller. How to compute invariant manifolds and their reduced dynamics in high-dimensional finite-element models? *Nonlinear Dyn.*, 2021.

[4] A. Logg, K.-A. Mardal, G.N. Wells, et al. *Automated Solution of Differential Equations by the Finite Element Method*. Springer, 2012.

[5] H.P. Langtangen and A. Logg. *Solving PDEs in Python*, volume 3 of *Simula SpringerBriefs on Computing*. Springer, 2016.

[6] J.-C. Loiseau, M.A. Bucci, S. Cherubini, and J.-C. Robinet. Time-stepping and Krylov methods for large-scale instability problems. In A. Gelfgat, editor, *Computational Modelling of Bifurcations and Instabilities in Fluid Dynamics*, pages 33–73. Springer International Publishing, Cham, 2019.

[7] P.J. Holmes, J.L. Lumley, G. Berkooz, and C.W. Rowley. *Turbulence, Coherent Structures, Dynamical Systems and Symmetry*. Cambridge Monographs on Mechanics. Cambridge University Press, 2 edition, 2012.

[8] K. Taira, S.L. Brunton, S.T.M. Dawson, C.W. Rowley, T. Colonius, B.J. McKeon, O.T. Schmidt, S. Gordeyev, V. Theofilis, and L.S. Ukeiley. Modal analysis of fluid flows: an overview. *AIAA Journal*, 55(12):4013–4041, 2017.

[9] B.R. Noack, K. Afanasiev, M. Morzyński, G. Tadmor, and F. Thiele. A hierarchy of low-dimensional models for the transient and post-transient cylinder wake. *Journal of Fluid Mechanics*, 497:335–363, 2003.

[10] S.L. Brunton, J.L. Proctor, and J.N. Kutz. Discovering governing equations from data by sparse identification of nonlinear dynamical systems. *Proceedings of the National Academy of Sciences*, 113(15):3932–3937, 2016.

[11] K. Fukami, T. Murata, K. Zhang, and K. Fukagata. Sparse identification of nonlinear dynamics with low-dimensionalized flow representations. *J. Fluid Mech.*, 926:A10, 2021.

[12] P.J. Schmid. Dynamic mode decomposition of numerical and experimental data. *J. Fluid Mech.*, 656:5–28, 2010.

[13] J.N. Kutz, S.L. Brunton, B.W. Brunton, and J.L. Proctor. *Dynamic Mode Decomposition*. SIAM, Philadelphia, PA, 2016.

[14] B. Bäuerlein and K. Avila. Phase lag predicts nonlinear response maxima in liquid-sloshing experiments. *J. Fluid Mech.*, 925, 2021.

[15] D. Dylewsky, E. Kaiser, S.L. Brunton, and J.N. Kutz. Principal component trajectories for modeling spectrally-continuous dynamics as forced linear systems. *arXiv:2005.14321*, 2021.

[16] X. Cabré, E. Fontich, and R. de la Llave. The parameterization method for invariant manifolds i: Manifolds associated to non-resonant subspaces. *Indiana Univ. Math. J.*, 52(2):283–328, 2003.

[17] R. Szalai, D. Ehrhardt, and G. Haller. Nonlinear model identification and spectral submanifolds for multi-degree-of-freedom mechanical vibrations. *Proc. Royal Society A*, 473(2202):20160759, 2017.

[18] S. Ponsioen, T. Pedergnana, and G. Haller. Automated computation of autonomous spectral submanifolds for nonlinear modal analysis. *J. Sound and Vibration*, 420:269–295, 2018.

[19] T. Breunung and G. Haller. Explicit backbone curves from spectral submanifolds of forced-damped nonlinear mechanical systems. *Proc. Royal Soc. A*, 474:20180083, 2018.

[20] S. Ponsioen, T. Pedergnana, and G. Haller. Analytic prediction of isolated forced response curves from spectral submanifolds. *Nonlinear Dyn.*, 98:2755–2773, 2019.

[21] S. Ponsioen, S. Jain, and G. Haller. Model reduction to spectral submanifolds and forced-response calculation in high-dimensional mechanical systems. *J. Sound and Vibration*, 488:115640, 2020.

[22] A. Haro, M. Canadell, J.-L. Figueras, A. Luque, and J.M. Mondelo. *The Parameterization Method for Invariant Manifolds: from Rigorous Results to Effective Computations*. Springer, New York, 2016.
